# Engineered gradient oxygen vacancies by ambient ball-milling boost ampere-level water electrolysis stability

**DOI:** 10.1093/nsr/nwag070

**Published:** 2026-02-09

**Authors:** Min Lu, Yang Hu, Shuhui Li, Xiong Liu, Li An, Yong Peng, Pinxian Xi, Chun-Hua Yan

**Affiliations:** Department of Inorganic Chemistry, College of Chemistry and Chemical Engineering, Frontiers Science Center for Rare Isotopes, Lanzhou University, Lanzhou 730000, China; Department of Materials Physics, School of Materials and Energy, Electron Microscopy Centre of Lanzhou University, Lanzhou 730000, China; Department of Inorganic Chemistry, College of Chemistry and Chemical Engineering, Frontiers Science Center for Rare Isotopes, Lanzhou University, Lanzhou 730000, China; Department of Inorganic Chemistry, College of Chemistry and Chemical Engineering, Frontiers Science Center for Rare Isotopes, Lanzhou University, Lanzhou 730000, China; Department of Inorganic Chemistry, College of Chemistry and Chemical Engineering, Frontiers Science Center for Rare Isotopes, Lanzhou University, Lanzhou 730000, China; Department of Inorganic Chemistry, State Key Laboratory of Applied Organic Chemistry, Lanzhou University, Lanzhou 730000, China; Department of Materials Physics, School of Materials and Energy, Electron Microscopy Centre of Lanzhou University, Lanzhou 730000, China; Department of Inorganic Chemistry, College of Chemistry and Chemical Engineering, Frontiers Science Center for Rare Isotopes, Lanzhou University, Lanzhou 730000, China; State Key Laboratory of Baryunobo Rare Earth Resource Researches and Comprehensive Utilization, Baotou Research Institute of Rare Earths, Baotou 014030, China; Department of Inorganic Chemistry, College of Chemistry and Chemical Engineering, Frontiers Science Center for Rare Isotopes, Lanzhou University, Lanzhou 730000, China; Beijing National Laboratory for Molecular Sciences, State Key Laboratory of Rare Earth Materials Chemistry and Applications, PKU-HKU Joint Laboratory in Rare Earth Materials and Bioinorganic Chemistry, Department of Inorganic Chemistry, College of Chemistry and Molecular Engineering, Peking University, Beijing 100871, China

**Keywords:** oxygen-vacancy distribution, mechanochemistry, ampere-level water electrolysis, structure–activity relationship

## Abstract

Balancing the activity and stability of catalysts has become one of the key approaches to breaking through the bottleneck of ampere-level water electrolysis. Oxygen vacancies are one of the most important active structures in catalysts. The complex structure of oxygen vacancies, such as the spatial distribution, profoundly affects the catalytic activity and mechanism. Here, we present a mechanochemical method that utilizes controlled environment pressure to create oxygen vacancies and modulate their spatial distribution in Pr_0.5_Ba_0.5_CoO_3_ by regulating the surface desorption and lattice-diffusion process of oxygen. Combining computational and experimental results, we propose a detailed synthesis mechanism that outlines the formation and diffusion behavior of vacancies at the atomic scale. Through further electrochemical studies, we develop two oxygen-evolution reaction descriptors—surface and bulk oxygen-vacancy concentrations—suitable for different catalytic mechanisms. Based on the above research, we further propose an oxygen-vacancy distribution-control strategy, which can significantly improve the stability of the catalyst at ampere-level current densities and under practical working conditions while maintaining their high intrinsic activity through the switching of catalytic mechanisms. This work offers a mechanochemical method for synthesizing oxygen vacancies and paves a new way for the development of ampere-level industrial electrolytic water catalysts.

## Teaser

This work develops a universal mechanochemical strategy for the spatial control of oxygen vacancies and establishes a relationship between vacancy distribution and the oxygen evolution reaction mechanism, enabling the synergistic enhancement of activity and stability for alkaline water electrolysis at industrial current densities.

## INTRODUCTION

Hydrogen serves as an ideal energy carrier, effectively addressing the pressing demand for clean energy in contemporary society [1,2]. Among various hydrogen-production methods, electrochemical water splitting powered by renewable energy, particularly through established alkaline electrolysis systems, offers notable advantages, including scalability, cost-effectiveness and environmental compatibility [3,4]. It is the core technical route towards a green hydrogen economy at the present stage. However, the oxygen-evolution reaction (OER) at the anode involves complex four-electron-transfer steps and high kinetic potential barriers, which result in relatively low overall energy-conversion efficiency [5,6]. This limitation has emerged as a critical bottleneck hindering large-scale industrial deployment. Over the years, many research efforts have been devoted to developing novel and efficient electrocatalysts to improve the OER performance, yielding significant progress [7–9]. However, the problem of the rapid degradation of activity due to catalyst-structure reconfiguration or collapse under long-term operation at high current densities (>500 mA cm^−2^, even reaching ampere levels) and strong corrosive conditions (80°C and 30% KOH solution) has become increasingly prominent. Under such harsh operating environments, intense electron diffusion and the strong oxidative environment exacerbate the instability of the electrocatalyst structure [10]. Conventional strategies for improving performance often face a trade-off between enhancing activity and maintaining structural integrity, as high catalytic activity is frequently accompanied by irreversible oxidation or dissolution of key active sites under high-potential conditions [11].

Among the various strategies for enhancing the performance of electrocatalysts, defect engineering (particularly the design of oxygen vacancies) demonstrates remarkable potential. Fundamentally, oxygen vacancies (V_O_) refer to the absence of oxygen atoms within the crystal lattice, which introduces unique structural and electronic features into the host material [12–14]. As localized charge modulation centers, V_O_ can effectively regulate the electron distribution around active sites, optimize the adsorption energy of reaction intermediates and thereby lower the energy barriers associated with rate-determining steps [15,16]. Furthermore, the introduction of dangling bonds by V_O_ significantly increases the intrinsic electrical conductivity, accelerating the charge-transfer process [17,18]. More importantly, V_O_ can either serve directly as active sites or modify the electronic configuration of neighboring metal centers, enabling the OER to switch between various mechanisms [18,19]. Despite these advantages, most conventional methods for synthesizing and controlling V_O_ exhibit notable limitations: they typically lack spatial selectivity in vacancy formation, leading to disordered and non-uniform distributions of V_O_ both in the near-surface region and within the bulk structure. This random spatial distribution gives rise to two major challenges.

Firstly, the local chemical environments of different V_O_ show significant differences, leading to inconsistent electronic structures and adsorption capabilities for reaction intermediates. This makes it difficult to rationally explain the catalytic process and guide catalyst design based on the structure–activity relationships established experimentally. Secondly, the disorderedly distributed V_O_ are more likely to undergo structural relaxation, annihilation or reoccupation by oxygen species under strong oxidizing conditions at high current density/high overpotential. These processes can directly lead to the structural collapse and deactivation of the catalysts, affecting their stability and operating lifetime. Therefore, precisely locating and regulating the distribution of V_O_ in catalysts has become the key strategy for achieving the synergistic optimization of activity and stability, and is also the bridge connecting basic defect research and industrial application.

In this work, we developed an environmentally controlled ball-milling method to successfully achieve the controllable construction of oxygen vacancies in Pr_0.5_Ba_0.5_CoO_3_ (PBCO). By regulating the Ar pressure in the ball-milling tank, we achieved spatial-distribution gradient control of the defect structure and visualized the distribution of V_O_ by using electron microscopy and depth-profiling techniques. We also demonstrated the universality of this method for the construction and distribution regulation of other types of anion defects in PBCO, such as the substitutional doping of sulfur. Density functional theory (DFT) calculations confirmed the energy trends of PBCO with different spatial distributions of V_O_, providing computational evidence of the formation mechanism and distribution differences of V_O_. Subsequently, by controlling the defect distribution, we achieved the switching of the OER mechanism and the balance of catalytic activity under the two mechanisms. We further established the structure–activity relationship between the V_O_ concentration on the surface or bulk and the catalytic activity under different mechanisms. Through this structure–activity relationship, we successfully demonstrated the promotion of a V_O_ distribution-control strategy on the activity and stability of large current water splitting under industrial conditions and achieved the stable operation of an alkaline electrolyser at an ampere-level current density. Our study highlights the critical role of the spatial distribution of V_O_ in modulating both the activity and the reaction mechanisms of the OER and various other catalytic processes. At the same time, we proposed a new strategy to address the contradiction between the activity and stability of catalysts in water splitting at an ampere-level current density. Additionally, our work provides new insights into the mechanochemical synthesis method for defect-controllable construction and fine structure regulation.

## RESULTS AND DISCUSSION

### Synthesis and structural characterization of PBCO catalysts with different oxygen vacancies

**Figure 1.**
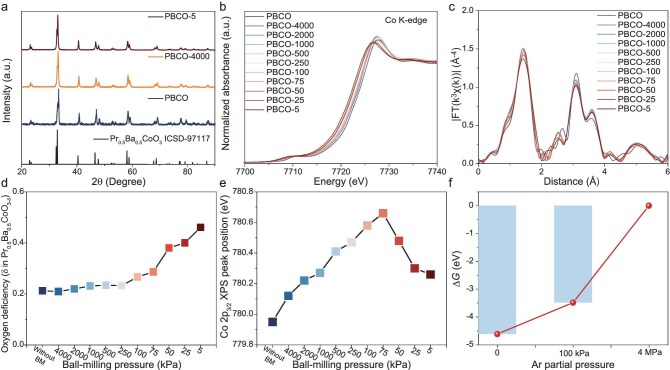
(a) X-ray diffraction (XRD) patterns of PBCO samples. (b) Co K-edge X-ray absorption near edge structure (XANES) spectra of all PBCO samples. (c) Co K-edge extended X-ray absorption fine structure (EXAFS) spectra of all PBCO samples. (d) Calculated oxygen vacancies content of PBCOs. (e) Binding energy of Co 2*p*_3/2_ XPS signal of all PBCOs. (f) DFT simulations of formation energy of V_O_ in the PBCO supercell under varying pressures.

The original PBCO sample was synthesized via the solid-state method [20]. High-temperature annealing was conducted to minimize the initial V_O_ concentration in the material as effectively as possible. Subsequently, we developed an environmentally controlled ball-milling method to precisely introduce V_O_ into the PBCO structure (Figs S1–S3). By modulating both the gas atmosphere and the pressure during the ball-milling process, we were able to quantitatively regulate the concentration and distribution of vacancies within the material while maintaining a constant mechanical impact intensity. As shown in Fig. 1a, Figs S4–S8 and Tables S1–S3, the perovskite structure, elemental composition and morphology of the materials are retained following ball-milling under different pressures. Furthermore, by maintaining consistent mechanical strength throughout the milling process, additional interference from mechanical process differences on the material pulverization process can be eliminated. It can further effectively eliminate additional interference from physical parameters such as particle-size and surface-energy differences in material research, thereby expanding the application scope and synthesis capability of mechanochemical synthesis methods.

The V_O_ structure was further investigated by using X-ray photoelectron spectroscopy (XPS) and X-ray absorption spectroscopy (XAS) (Fig. 1b–e, Fig. S9 and Table S4). We quantitatively characterized the oxidation state of Co in PBCOs through XAS (Fig. 1b and Fig. S9). Based on the oxidation states of other metals and the principle of charge neutrality, we further calculated the total (bulk) content of V_O_ in PBCOs (Fig. 1d). The results reveal that the stoichiometric number of V_O_ in the original PBCO (without ball-milling) is ∼0.21 and the vacancy concentration is ∼7%. After the high-pressure ball-milling process (the pressure of Ar ranges from 4000 to 250 kPa), the V_O_ content in the bulk showed minimal variation when compared with that in the primary PBCO. Upon further reducing the Ar pressure within the ball-milling chamber to a vacuum state (from 250 to 5 kPa), the content and concentration of V_O_ increased approximately linearly from ∼0.23 and 7.7% to 0.46 and 15.3%, respectively. The extended X-ray absorption fine structure (EXAFS) results (Fig. 1c) further confirm that the introduction of V_O_ does not alter the local coordination environment, but selectively affects the Co–O coordination number.

On the other hand, due to the lack of quantitative techniques for surface defects, we conducted a semi-quantitative comparison of V_O_ concentrations on the surface. The variation in the binding energy of Co can reflect the differences in the cobalt valence states on the PBCO surface [21,22]. As shown in Fig. 1e and Fig. S10, as the Ar pressure decreases, the position of the Co 2*p*_3/2_ peak first moves approximately linearly towards higher binding energy and then decreases. PBCO-75 is the inflection point of the peak position variation. The above result indicates that the content of V_O_ on the PBCO surface exhibits a volcano-shaped relationshipwith the Ar pressure during synthesis.

The above results indicate that our environmentally controlled ball-milling method can simultaneously control both the bulk and surface V_O_ in PBCO materials. It also means that we have successfully manipulated the spatial-distribution gradient of V_O_. DFT results also demonstrated that the pressure of the Ar affects the formation energy of V_O_ in the material (Fig. 1f). The vacuum environment promotes the generation of V_O_, while higher pressure inhibits this process. The observed contrast between the higher bulk V_O_ levels and the lower surface V_O_ concentration implies that the sustained rise in the surface V_O_ density promotes the accelerated diffusion of vacancies from the surface into the bulk. This diffusion of V_O_ is driven by both the chemical potential difference and mechanical energy, which can prevent the structural collapse and amorphization caused by the accumulation of defects on the surface and reduce the energy barrier for the continuous generation of V_O_.

### Spatial-distribution gradient of oxygen vacancies

**Figure 2.**
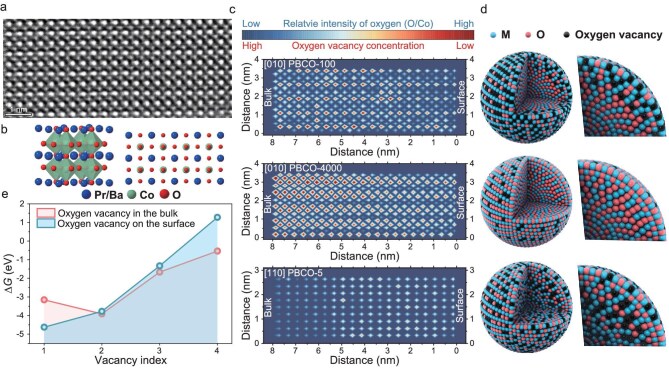
(a) Annular bright-field scanning transmission electron microscopy (ABF-STEM) image of PBCO-100. (b) Crystal structure of PBCO (left) and the atomic arrangement pattern corresponding to the ABF-STEM atomic image in (a) (right). (c) Intensity-distribution map of oxygen extracted from Fig. 2a, and Figs S12 and S14. (d) Distribution model of V_O_ derived from the intensity-distribution map of oxygen atom columns in (c). (e) Associated Δ*G*_vfb_ and Δ*G*_vfs_ for PBCO with varying vacancy concentrations across different regions.

To gain deeper insight into the distribution variation of V_O_ within PBCO, we performed annular bright-field scanning transmission electron microscopy (ABF-STEM). We carefully obtained the ABF images of the surface of PBCO-100 particles. From the ABF images, the atomic arrangement corresponds to the [010] direction of Pr_0.5_Ba_0.5_CoO_3_ (Fig. 2a and b). Following this, the intensity of individual oxygen- and cobalt-atom columns was extracted from the ABF image and the relative intensity ratio of O/Co was calculated by comparing each oxygen column with its neighboring cobalt columns. This approach was used to minimize the influence of the sample thickness and other extraneous factors (Fig. 2c and d, and Figs S12 and S13). The results show that the O-atom column on the surface of PBCO-100 exhibits the lowest relative intensity, indicating the enrichment of V_O_. Furthermore, from surface to bulk, the relative intensity of O gradually increases, reaching its maximum within a range of ∼4–6 nm, and then decreases. This suggests that the V_O_ concentration initially decreases and subsequently rises. We also conducted the same analysis on PBCO-5 and PBCO-4000 (Fig. 2c and d, and Figs S14–S17). In PBCO-4000, V_O_ are predominantly located at the surface, with their concentration gradually decreasing from the surface toward the bulk. In contrast, PBCO-5 exhibits a primary distribution of V_O_ within the bulk. In a selected ABF region, within the surface range of ∼0–2 nm, the V_O_ concentration shows a slight decrease and then gradually increases in the range of 2–8 nm.

XPS depth profile analysis (Figs S18 and S19) and detailed X-ray diffraction (XRD) analysis (Figs S20 and S21) were conducted to further substantiate the V_O_ distribution obtained via ABF. As shown in Fig. S19, with increasing etching time and depth, the concentration changes of the surface V_O_ across different materials display significant differences. In PBCO-4000, the concentration steadily declines from the surface toward the interior, suggesting a predominant surface localization of vacancies. For PBCO-5, the concentration of V_O_ initially decreases and then increases. This indicates that V_O_ in PBCO-5 are distributed almost uniformly in the bulk. In comparison, PBCO-250 and PBCO-100 show an initial reduction in V_O_ concentrations close to the surface, followed by a temporary rise before a sustained decrease at larger depths. It shows that the V_O_ mainly accumulate at the subsurface.

The XRD results further reveal the changes in the distribution of V_O_ caused by diffusion from the perspective of the crystal structure (Figs S20 and S21). After internal standard correction, the positions of the major diffraction peaks ((110), (102) and (112)) of the material showed minimal variation from PBCO-4000 to PBCO-250. This indicates that, within the detection depth of XRD, the average lattice parameters of the materials did not undergo significant changes [23–25]. This phenomenon occurs because V_O_ located on the particle surface give rise to local lattice distortions, which can be masked by the strong signal from the bulk lattice. However, when the vacancies are distributed over a wide region, lattice distortion can be detected by XRD. The positive shift in the diffraction peaks observed from PBCO-250 to PBCO-5 and the lattice contraction indicate that the V_O_ in these materials are gradually diffusing from the surface into the bulk. The aforementioned results align closely with the ABF results, confirming that the distribution of V_O_ in PBCOs has been effectively controlled through ball-milling.

Additional DFT simulations were carried out to explore the formation energy of V_O_ with varying distributions in PBCO (Figs S22–S25). Oxygen atoms were randomly removed from either the surface or the interior of PBCO to model surface and bulk V_O_ formation, respectively. The corresponding energies are referred to as the Gibbs free energy of the V_O_ formation process on the surface (Δ*G*_vfs_) and in the bulk (Δ*G*_vfb_), respectively [26–29]. Notably, at low V_O_ concentrations, the surface V_O_ exhibits a lower formation energy (−4.62 eV) compared with that of the bulk V_O_ (−3.16 eV). However, with increasing V_O_ concentration, the formation energy of surface V_O_ progressively becomes higher than that of the bulk V_O_. This suggests that a high density of surface V_O_ can destabilize the lattice structure. The calculation of V_O_ migration energy further reveals that, at higher V_O_ concentrations, the vacancies will thermodynamic-spontaneously migrate from the surface to the bulk. We further carried out an in-depth analysis of the V_O_–V_O_ interactions during the formation process of V_O_. As shown in Fig. S25a, when the second oxygen vacancy is located at the *meta*-position, *ortho*-position or opposite-position to the first oxygen vacancy, the formation energy of V_O_ exhibits significant differences, at −3.08, −3.54, and −3.78 eV, respectively. As V_O_ carry two forms of positive charges, there is an electrostatic repulsion between the V_O_. The above results clearly demonstrate that the formation energy of the second V_O_ is positively correlated with the distance between the V_O_. That is, the closer the distance between the two V_O_, the more difficult it is to form. This result also explains the observed differences in V_O_ concentration and distribution. When the concentration of the surface V_O_ increases, the electrostatic repulsion between them also increases, thereby inhibiting further generation of surface V_O_ and causing them to diffuse to the more distant bulk region. As a result, the V_O_ in the surface tend to diffuse into the bulk thermodynamically to lower the overall internal energy and enhance the structure stability.

### Synthesis mechanism of oxygen vacancies in the mechanochemical process

**Figure 3.**
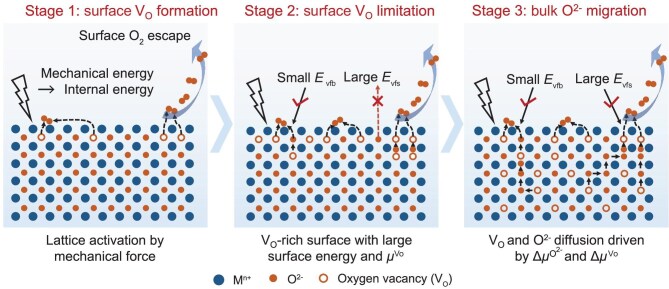
Synthesis mechanism of V_O_ in the mechanochemical process.

Based on the above results, we propose the microscopic mechanism of oxygen-vacancy synthesis by a mechanochemical process (Fig. 3). During the atmosphere-controlled ball-milling process, the external gas pressure determines the generation location, diffusion behavior and spatial-distribution configuration of V_O_ through the simultaneous regulation of the thermodynamic and kinetic behavior of mechanochemical reactions. In thermodynamics, the shear, friction and impact forces generated by ball-milling first act on the particle surface, activating the surface lattice and reducing the energy barrier for the release of surface O^2−^, thereby favoring the generation of surface V_O_. In kinetics, increased Ar pressure will lead to an increase in the number of Ar molecules per unit volume. It will subsequently enhance the frequency of molecular collisions and further inhibit the desorption of adsorbed O_2_ on the surface, thereby inhibiting the formation of V_O_.

In the initial stage (Stage 1), because mechanical forces restrict the initial formation area of vacancies, V_O_ are created on the surface of the material and then cause the surface energy to rise. At this stage, the V_O_ concentration on the surface of the material is very small and the bulk concentration is basically the same. So, the V_O_ diffusion tendency to the bulk is also small. In that case, V_O_ form and aggregate on the surface of the material. When the V_O_ concentration of the surface increases continuously, the difference between the V_O_ concentrations of the surface and of the bulk also increases. This causes a chemical potential gradient of oxygen to appear at different depths of the material lattice and drives O^2−^ to diffuse from the bulk to the surface along the oxygen-vacancy sites. It also means the diffusion of V_O_ from the surface to the bulk. The continuous mechanical forces acting on the surface of the material provide diffusion energy for this process.

On the other hand, the formation of surface V_O_ will destabilize the surface structure and increase the surface energy and surface V_O_ formation energy (*E*_vfs_). This also reduces the formation rate of surface V_O_ (*v*_vfs_) and increases the diffusion rate of surface V_O_ to the bulk (*v*_vfb_). This results in a limit to the V_O_ concentration on the surface. Then, the formation mechanism of V_O_ reaches Stage 2. The high V_O_ concentration on the surface makes it difficult to form further V_O_ and the high chemical potential difference between the surface and the bulk significantly increases the *v*_vfb_.

With the transition from Stage 2 to Stage 3, the surface V_O_ diffuse rapidly to the bulk. Currently, because the defect-rich surface still has a high surface energy, the diffusion rate is greater than the formation rate of V_O_, resulting in a decrease in the surface V_O_ concentration. Then, the reduced surface energy and *E*_vfs_ allow the surface to continue to form V_O_. On the other hand, driven by the chemical potential difference, V_O_ continues to diffuse to the bulk and tends to be evenly distributed in the whole material.

### Universality of the defect distribution regulation method

Sulfur and oxygen belong to the same group of elements. Sulfur ions (S^2−^) and oxygen ions (O^2−^) share many similarities. In perovskite-like oxide, S^2−^ can partially replace O^2−^ in the lattice, forming substitute sulfur defects [30–32]. The method we developed for regulating oxygen-vacancy distribution is based on the directional diffusion of ions in solids. Therefore, we can predict that this method can also introduce substitutionally doped S^2−^ into oxides and control their spatial distribution.

The XRD results (Fig. S26) show that, after being ball-milled together with sulfur, all PBCO materials still retained their original perovskite structures. On the other hand, no sulfur powder or solid was observed after the CS_2_ washing solution dried. The above results indicate that the introduced sulfur was likely doped into PBCO without altering the crystal structure of the perovskite or inducing a phase transition in the material. From the S 2*p* XPS results (Fig. S27), the M–S and S–O components can be clearly identified, indicating that the doped sulfur has entered the perovskite oxide lattice and occupied the oxygen sites [33,34]. We used SEM-EDX to characterize the relative content of S/Co in the materials. The results (Fig. S28) showed that the S/Co atomic ratio in all samples was basically the same and was higher than the feed ratio (10 mol%). This result might be due to the fact that the detection depth of SEM-EDX cannot cover all of the material particles. That is to say, the sulfur dopant is present in particles with a concentration gradient and surface enrichment.

Further Co K-edge X-ray absorption fine structure (XAFS) characterization results (Fig. S29) show that both the Co valence state and the oxygen-vacancy content exhibit discontinuous two-stage changes with Ar pressure. First, the total V_O_ contents of the materials ranging from PBCO-S-4000 to PBCO-S-250, synthesized with a milling pressure of >100 kPa, are 0.332, 0.342, 0.341, 0.336 and 0.324, respectively, exhibiting a numerical fluctuation range of <0.02. It can be reasonably concluded that the total oxygen defect content across these five samples remains essentially unchanged. When the milling pressure is further reduced from 100 to 5 kPa, both the Co valence state and the total V_O_ contents exhibit significant changes. As the pressure continues to decrease, the V_O_ content gradually rises and reaches a maximum at 50 kPa and then remains stable. The total V_O_ contents of PBCO-S-100 to PBCO-S-5 are 0.532, 0.550, 0.567, 0.573 and 0.570, respectively.

Based on the synthesis mechanism of oxygen vacancies in the mechanochemical process, we speculate that, when there is S in the system, S^2−^ may exchange with O^2−^ or fill the oxygen-vacancy sites. The substituted S^2−^ ions subsequently migrated and diffused into the bulk. This diffusion process is analogous to the diffusion of V_O_ into the bulk or the diffusion of O^2−^ to the surface. The diffusion of S^2−^ is driven by concentration gradients and facilitated by mechanical energy activation to stabilize the lattice structure.

Subsequent characterization of the S^2−^ distribution confirmed the above mechanism. XPS and SEM-EDX were employed to analyse the sulfur concentrations at the surface and in the bulk (Fig. S30). PBCO-S-4000 to PBCO-S-1000 exhibit the highest surface S content, with an S/Co atomic ratio of ∼0.33. The surface S content of those materials is much higher than the S content in the bulk, as given by SEM-EDX (∼0.15) and the actual feeding ratio (∼0.1). When the Ar gas pressure is <1000 kPa, the surface S content begins to decrease. Within the Ar pressure range of 500–25 kPa, the S/Co ratio drops from 0.31 to 0.23, showing a nearly linear decrease. When the Ar pressure is further reduced to 5 kPa, the S/Co ratio acceleration drops to ∼0.15. For PBCO-S-5, the surface S content is consistent with the bulk S content. These phenomena indicate that, under high-pressure conditions, the doped sulfur is enriched at the surface. As environmental pressure decreases, the S^2−^ gradually diffuses into the bulk. The above results provide direct evidence for the proposed synthesis mechanism.

The diffusion of S^2−^ into the bulk lattice essentially involves an exchange between S^2−^ in the near-surface region and V_O_/O^2−^ in the deeper layers. Lower environmental pressures promote the outward diffusion of bulk O^2−^ to the surface and enable V_O_ sites to diffuse inward, thereby creating diffusion pathways that facilitate deeper penetration of S^2−^ into the lattice (Fig. S31).

This clear trend in the surface sulfur concentration changes offers robust experimental support for understanding the coupled dynamics of oxygen-ion and oxygen-vacancy diffusion within the material lattice. More importantly, using S as a labeling atom, the above results further prove the spatial-distribution regulation mechanism of V_O_ based on ion diffusion that we previously proposed.

### Correlation between vacancy distribution and OER performance

**Figure 4.**
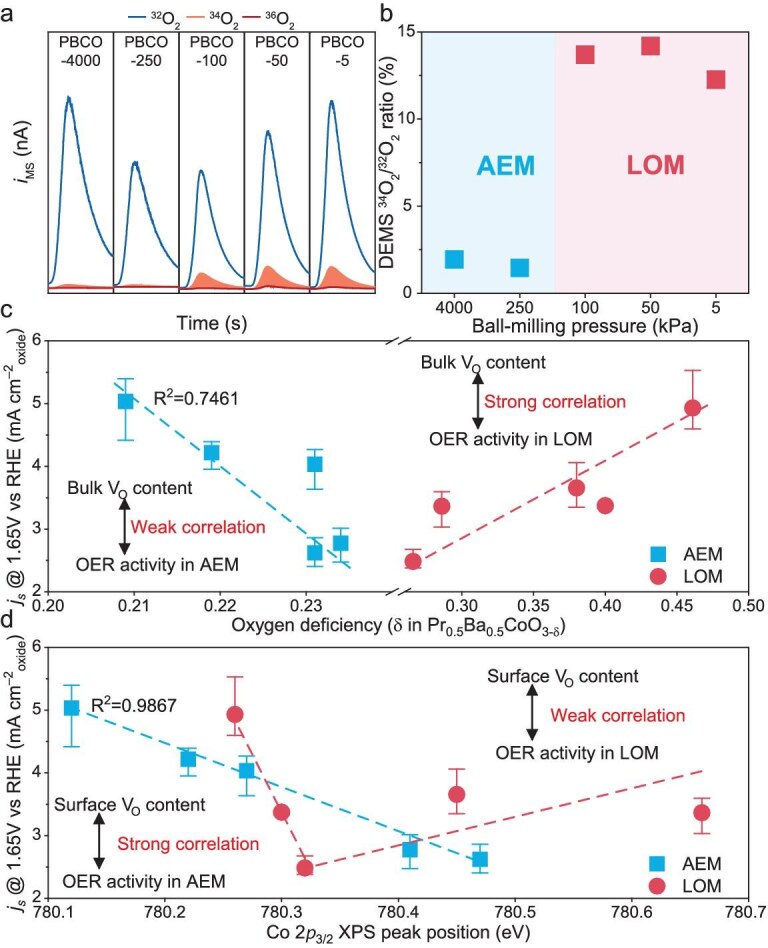
(a) DEMS signals of ^36^O_2_ (^18^O^18^O), ^34^O_2_ (^16^O^18^O) and ^32^O_2_ (^16^O^16^O) from the OER reaction products for ^18^O-labeled PBCO-4000, PBCO-250, PBCO-100, PBCO-50 and PBCO-5. (b) DEMS signals ratio of ^34^O_2_ (^16^O^18^O) and ^32^O_2_ (^16^O^16^O) from the reaction products for ^18^O-labeled PBCO catalysts test in H_2_^16^O aqueous KOH electrolyte. (c) Correlation between activities/mechanisms of OER and concentration of bulk V_O_ in PBCOs. (d) Correlation between activities/mechanisms of OER and concentration of surface V_O_ in PBCOs.

To evaluate the intrinsic OER activity of PBCO catalysts, cyclic voltammetry (CV) measurements were performed. After normalizing the current density by the Brunauer-Emmett-Teller (BET) surface area of each sample, it is evident from Fig. S35 that reducing the Ar pressure during synthesis causes the intrinsic OER activity of the material to exhibit a V-shaped pattern. Two primary reaction mechanisms were identified for the OER process. Subsequently, we characterized the dominant mechanism by using differential electrochemical mass spectroscopy (DEMS) coupled with ^18^O labeling (Fig. 4a and b). PBCO-4000 and PBCO-250, which exhibit varying surface V_O_ concentrations but nearly identical bulk V_O_ levels, produce negligible amounts of ^18^O signals, indicating an adsorbate evolution mechanism (AEM) [35,36]. In contrast, PBCO-100, PBCO-50 and PBCO-5 generate 13.7%, 14.2% and 12.3% ^34^O_2_, respectively, which supports the presence of a characteristic lattice oxygen oxidation mechanism (LOM). Furthermore, PBCO-250 and PBCO-100 mark the transition region between the AEM and the LOM, coinciding with a notable shift in the bulk V_O_ concentration. Therefore, as shown in Fig. S37, the concentration parameters of bulk V_O_ can serve as a mechanism descriptor.

The correlation between OER activity and the concentrations of V_O_ in both the bulk and surface across different OER mechanisms was deeply examined, as shown in Fig. 4c and d. Under the AEM, the OER activity shows a strong linear relationship with the surface V_O_ concentration (*R*^2^ = 0.9867, Fig. 4d), which is significantly higher than that with the bulk V_O_ concentration (*R*^2^ = 0.7461, Fig. 4c). This phenomenon can be explained by the nature of the AEM–OER mechanism, in which catalytic intermediates participate exclusively in surface adsorption, desorption and conversion processes. As a result, the OER activity is closely tied to the electronic configuration of the surface active sites. In contrast, the LOM involves the oxidation of lattice oxygen within the bulk material, leading to a strong dependence of the LOM–OER activity on the bulk V_O_ concentration (Fig. 4c), while showing no notable relationship with the surface V_O_ levels (Fig. 4d). Therefore, the bulk V_O_ content acts as a key descriptor for both the OER mechanism and the LOM–OER performance, whereas the surface V_O_ concentration specifically serves as a descriptor for the AEM–OER activity.

We further conducted a detailed analysis of the intrinsic relationship between the spatial distribution of V_O_ and the OER mechanism/activity. During the OER process, the fundamental difference between the AEM and the LOM lies in whether the reaction is limited to the surface adsorption intermediates or further utilizes the bulk lattice oxygen to participate in bonding and oxygen release [37].

When the V_O_ are mainly distributed on the surface, their main function is to regulate the electronic configuration of the surface Co active sites and optimize the adsorption/desorption energies of reaction intermediates (such as OH* and OOH*). In this situation, the crystal structure remains at a complete stoichiometric ratio and the lattice structure is relatively rigid. The active Co sites maintain a strong Co–O bond strength with the lattice oxygen ligands. This high stability prevents the lattice oxygen from participating in the reaction, forcing the reaction to follow the traditional AEM–OER. On the contrary, when the V_O_ are largely distributed in the bulk, the covalent nature of the Co–O bonds in the lattice increases (Fig. S25) and the O^2−^ diffusion ability also improves. This makes it easier for the oxygen in the lattice to be oxidized through electrochemical reactions and directly participate in the formation of the O–O bond. At this time, V_O_ not only provide channels for oxygen diffusion, but also reduce the energy barrier for the OER to transform into the LOM. Under this mechanism, the activity is more dependent on the concentration of the bulk V_O_, while the correlation with the surface V_O_ is weakened. The calculation of the OER free energy and the rate-limiting step also confirmed the above conclusion (Fig. S38). When the V_O_ are located on the surface of the PBCO supercell, the energy barrier for the AEM–OER is lower, whereas, when the V_O_ are distributed in the bulk of the PBCO supercell, the energy barrier for the LOM–OER is lower.

### Dynamic structural evolution during the OER

**Figure 5.**
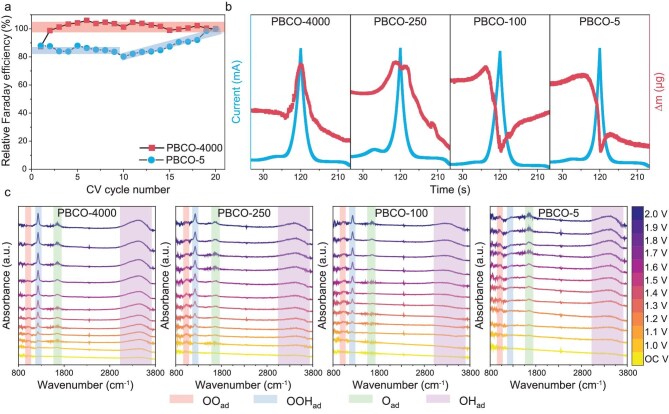
(a) Relative Faraday efficiency variation curve of PBCO-4000 and PBCO-5 during OER obtained from the 20-cycle DEMS results. (b) Electrochemical quartz crystal microbalance data from the initial CV cycle of PBCO-4000, PBCO-250, PBCO-100 and PBCO-5. (c) *In situ* IR results of PBCO-4000, PBCO-250, PBCO-100 and PBCO-5 under different voltages.

DEMS can semi-quantitatively monitor the changes in the O_2_ content of the OER and allows estimation of the Faraday efficiency by analysing the relative changes between the O_2_ content and the electrochemical parameters. *In situ* DEMS results reveal that the Faraday efficiency of the OER remains nearly constant over 20 CV cycles for PBCO-4000 (Fig. 5a), indicating structural stability of the catalyst. However, PBCO-5, which follows the LOM, undergoes notable surface oxidation and reconfiguration during the OER, leading to a consistently lower Faraday efficiency (<90%) in the initial stages of the reaction. These findings confirm that the AEM contributes to enhanced catalytic stability.

Electrochemical quartz crystal microbalance enables the precise tracking of mass changes resulting from surface reconstruction and surface adsorption or desorption of intermediates during electrochemical processes (Fig. 5b). During the OER, the mass of PBCO-250 and PBCO-4000 rises as the applied voltage increases and decreases correspondingly when the voltage is lowered. These mass variations are primarily attributed to the dynamic equilibrium between the adsorption and desorption of catalytic intermediates [38,39]. The more pronounced mass increase observed for PBCO-4000 corresponds to its higher OER activity.

In contrast, PBCO-5 and PBCO-100 display a slight mass increase before the onset potential of the OER, which is likely due to the oxidation of metal sites and the partial reduction of V_O_ under oxidative potentials, stemming from their initially high V_O_ content [40]. During the OER, the mass of these samples decreases with increasing potential, indicating the participation of lattice oxygen in the LOM–OER. This lattice oxygen involved in the reaction contributes to O_2_ formation at the surface and subsequently escapes from the crystal structure [40]. The mass recovery observed during the voltage-reduction stage indicates the dynamic balance between the formation and restoration of V_O_.

Due to the surface-sensitive nature, *in situ* attenuated total reflection infrared spectroscopy is commonly used to study the adsorption behavior of catalytic reaction intermediates on material surfaces during electrochemical processes (Fig. 5c). The OER mechanisms of PBCO-4000 and PBCO-250 are predominantly governed by the AEM. Characteristic infrared absorption peaks correspond to OO_ad_, OOH_ad_, O_ad_ and OH_ad_ adsorption species, which are located at ∼1030, ∼1230, 1650 and ∼3420 cm^−1^, respectively. Compared with PBCO-250, PBCO-4000 shows stronger OOH_ad_ and OH_ad_ peaks under the OER voltage, suggesting a greater tendency to form OOH intermediates and higher catalytic activity. The significantly weakened OO_ad_ peak implies that this material is conducive to the rapid transformation and desorption of OO_ad_ to O_2_ during the OER process. For PBCO-100 and PBCO-5, the significantly weakened OOH_ad_ peak at the OER voltage indicates a transition of the dominant OER mechanism from the AEM to the LOM [41–43].

### Ampere-level water electrolysis performance under industrial conditions

**Figure 6.**
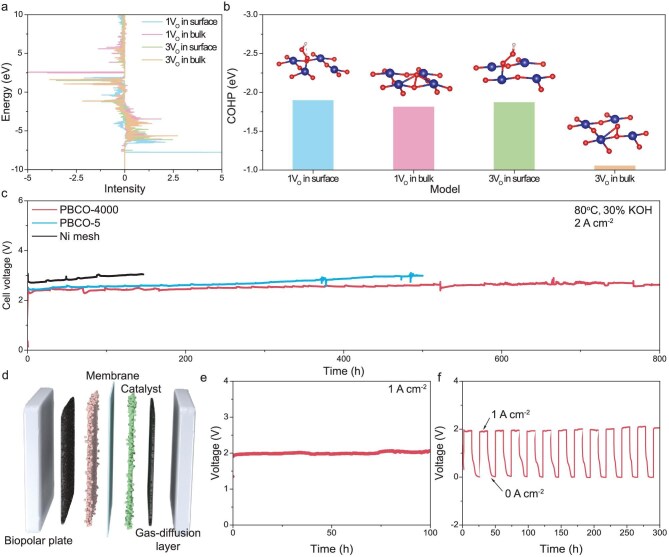
(a) COHP energy patterns of four different models calculated from DFT. (b) COHP analysis of Co–O bond for adsorbed oxygen-containing intermediate on four different models. (c) Two-electrode water electrolysis stability tests for PBCO-4000, PBCO-5 and pure nickel mesh at a current density of 2 A cm^−2^. (d) Schematic diagram of the alkaline electrolysis water device. (e) Water electrolysis performance of PBCO-4000 as the electrode material, with an electric current density of 1 A cm^−2^. (f) Voltage fluctuation resistance performance of water electrolysis with PBCO-4000 as the electrode material.

The above results demonstrate that it is possible to significantly enhance catalyst stability without compromising its high intrinsic activity by precisely controlling the vacancy distribution and switching the catalytic mechanism. To validate this strategy, we conducted water electrolysis measurements under operational conditions and ampere-level current density. The crystal orbital Hamilton population (COHP) of the surface adsorption sites for the four models during the OER was calculated using DFT. The results (Fig. 6a and b) show that, when a single oxygen vacancy exists on the surface and the OER follows the AEM, the COHP between the active Co site and the ligand O in the lattice reaches the most negative value, reflecting the strongest and most stable Co–O bond. Switching the catalytic mechanism to the LOM or increasing the V_O_ concentration significantly reduces the stability of the Co–O bond on the catalyst surface. The water electrolysis stability test under actual conditions (80°C, 30% KOH solution) and high current density (2 A cm^−2^) was further performed (Fig. 6c). The initial voltages of PBCO-4000, PBCO-5 and Ni mesh were 2.41, 2.44, and 2.71 V, respectively. The results show that, although PBCO-4000 and PBCO-5 exhibit different OER mechanisms, their water electrolysis activity was basically the same. More importantly, due to the suppression of large-scale surface reconstruction by the AEM, PBCO-4000 exhibited outstanding stability over 800 hours, whereas PBCO-5 underwent significant surface reconstruction, accompanied by a notable voltage increase after ∼300 hours. The corresponding termination voltages of PBCO-4000, PBCO-5 and Ni mesh are 2.57 V (800 h), 2.99 V (500 h) and 3.04 V (140 h). The voltage decay rates are 0.2, 1.1 and 2.4 mV/h, respectively. Finally, PBCO-4000 was integrated into a membrane electrode for alkaline water electrolysis. Under operating conditions and a current density of 1 A cm^−2^ with a cell voltage of 1.96 V and a voltage decay rate of 0.7 mV/h, the catalyst exhibited a lower operating voltage, excellent stability and strong resistance to voltage fluctuations (Fig. 6d–f).

## CONCLUSIONS

We have effectively realized the precise construction of V_O_ in Pr_0.5_Ba_0.5_CoO_3_ by employing a mechanochemical ball-milling process under regulated gas pressure conditions. By adjusting the Ar pressure within the milling environment, the concentration and spatial arrangement of V_O_ in the surface and the bulk of PBCO can be effectively modulated. Advanced characterization techniques, including ABF-STEM, depth profiling and detailed crystallographic analysis, confirm the diffusion of V_O_ from the surface into the bulk of the material. DFT simulations further elucidate the V_O_ distribution behavior by analysing their formation energies. Combining experimental and theoretical results, we establish a synthesis mechanism for V_O_ during mechanochemical treatment. Further electrochemical investigations have established the parameters of surface and bulk V_O_ concentrations as two key activity descriptors, which correspond to different OER mechanisms and reflect the underlying microscopic features of the reaction pathways. These results contribute to a deeper understanding of the fundamental connection between the vacancy structure and the oxygen-evolution process. Based on the above results, we achieved a balance in catalytic activity between the AEM and the LOM by modulating the distribution of V_O_. Furthermore, by switching the OER mechanisms, we significantly improved the stability of the material under operational conditions and at high current densities. The catalyst was then implemented in alkaline water electrolysis systems, where it exhibited a low operating voltage, outstanding long-term stability and robust tolerance to voltage fluctuations at a current density of 1 A cm^−2^. These results present a new perspective for understanding the origins of OER activity and mechanisms by precisely engineering the spatial distribution of V_O_. This work also proposes a novel approach for designing advanced catalysts suitable for ampere-level water electrolysis through precise control over the distribution of V_O_.

## Supplementary Material

nwag070_Supplemental_File
