# Supplementary material for: Engineered gradient oxygen vacancies by ambient ball-milling boost ampere-level water electrolysis stability
Source: Natl Sci Rev. 2026 Feb 9;13(6):nwag070. doi: 10.1093/nsr/nwag070 (PMC13045681; doi:10.1093/nsr/nwag070)
Supplement: nwag070_Supplemental_File [file nwag070_supplemental_file.pdf]

Supporting Information

Engineered gradient oxygen vacancies by ambient ball-milling boost ampere-level water electrolysis stability

Min Lu[1,†], Yang Hu[2,†], Shuhui Li[1,†], Xiong Liu[1], Li An[1,3], Yong Peng[2], Pinxian Xi[1,4,*] and Chun-Hua Yan[1,5,*]

[1]College of Chemistry and Chemical Engineering, Frontiers Science Center for Rare Isotopes, Lanzhou University, Lanzhou 730000, China.

[2]School of Materials and Energy, Electron Microscopy Centre of Lanzhou University, Lanzhou 730000, China.

[3]State Key Laboratory of Applied Organic Chemistry, Lanzhou University, Lanzhou 730000, China.

[4]State Key Laboratory of Baryunobo Rare Earth Resource Re-searches and Comprehensive Utilization, Baotou Research Institute of Rare Earths, Baotou 014030, China.

[5]Beijing National Laboratory for Molecular Sciences, State Key Laboratory of Rare Earth Materials Chemistry and Applications, PKU-HKU Joint Laboratory in Rare Earth Materials and Bioinorganic Chemistry, College of Chemistry and Molecular Engineering, Peking University, Beijing 100871, China.

*Corresponding authors. E-mails:xipx@lzu.edu.cn; yan@pku.edu.cn.

† These authors contributed equally.

Experimental Section

Materials: Pr_6O_{11}(99.9%), $BaCO_3$(99.99%), Co_3O_4(99.9%), NaOH (99%), KOH (99.99%) were purchased from Aladdin, Nafion® perfluorinated resin solution in lower aliphatic alcohols and water (5 wt%) was purchased from Sigma-Aldrich, absolute ethanol and isopropanol were purchased from Aladdin, the demonized (DI) water was obtained from a Millipore Auto-pure system (18.2 MΩ, Millipore Ltd., (USA). All other materials for electrochemical measurements were of analytical grade without further purification.

Synthesis of bulk $Pr_{0.5}Ba_{0.5}CoO_3$: PBCO bulk powders were synthesized by using a solid-state method from high-purity oxides. Stoichiometric amounts of Pr_6O_{11}, $BaCO_3$, and Co_3O_4 were thoroughly mixed by ball-milling (2 h ball-milling in PTFE milling tank, rotate speed is 500 rpm). Then the powder is heated to 1273 K for 12 hours at a heating rate of 2 K/min in air. The calcined product is ball-milled for 2 h under the same milling condition as above and then heated to 1373 K for 24 hours at a heating rate of 2 K/min in pure O_2.

Ball-milling treatment of bulk $Pr_{0.5}Ba_{0.5}CoO_{3-\delta}$: The ball-milling process was performed on an SFM-1 Planetary Ball-Mill machine purchased from Hefei Kejing Materials Technology Co. Ltd. Each sample was independently ball-milled. In a typical procedure, 1 g PBCO bulk powders were mixed with Φ 6 mm agate ball with a ball-to-powder ratio of 10:1 in the Ar filled glove box (O_2 concentration < 1ppm) and then sealed in a homemade high-pressure ball-milling container. The milling container is composed of a 130 mL PTFE inner container and a stainless-steeled outer shell. Subsequently, the air inlet of the milling container was connected to the argon gas cylinder through a high-pressure gas pipeline system. The air outlet was connected to a vacuum pump. The gas in the ball-milling container and the gas line was extracted through a vacuum pump, and then high-purity Ar gas (99.999%) was introduced. This process was repeated 10 times to ensure a high-purity argon environment throughout the entire system. Finally, fill the milling container with Ar until the desired pressure is reached. Then stop the gas filling, close the valve,

disconnect the milling container from the gas pipeline, and transfer it to the planetary ball mill machine for ball-milling. Then the ball-milling was performed at a Rotation speed and revolution speed of 400 rpm and 200 rpm for 10 h.

Synthesis of $Pr_{0.5}Ba_{0.5}CoO_{2.9-\delta}S_{0.1}$: The ball-milling process was performed on an SFM-1 Planetary Ball-Mill machine purchased from Hefei Kejing Materials Technology Co. Ltd. 1.23 g PBCO bulk powders and 16.0 mg S powders were mixed with Φ 6 mm agate ball with a mass ratio of 1:10 in the Ar filled glove box (O_2 concentration < 1 ppm) and then sealed in a homemade high-pressure ball-milling container. The ball-milling container is then flushed 10 times with Ar and then adjusted to a specific pressure by vacuuming or pressurizing with Ar. Then the ball-milling was performed at a rotational velocity of 400 rpm for 10 h. Subsequently, the product was washed multiple times with CS_2 via centrifugation and then dried to remove unreacted elemental sulfur.

Physicochemical Characterizations: XRD measurements were carried out on Rigaku MiniFlex 600 diffractometer with Cu Kα radiation (λ = 0.1542 nm) from 10° to 80 ° under a constant voltage of 40 kV. The morphology and EDX elemental mappings of samples were analyzed by Thermo Fisher Apreo S field-emission scanning electron microscope (FESEM) at an acceleration voltage of 30 kV. Elemental mappings were performed on Tecnai G2 F30 Field Emission Transmission Electron Microscopy. Atomic-scale STEM images were recorded on a probe aberration-corrected STEM (FEI Cubed Themis Z, FEI, USA) operated at 300 kV. Inductively coupled plasma optical emission spectrometry (ICP-OES) analyses were performed on a Plasma Quant PQ9000 ICP spectrometer. Synchrotron radiation X-ray absorption fine structure spectroscopy at the Co K-edge was acquired in transmission mode by using a Si (111) double-crystal monochromator at the 1W1B station of the Beijing Synchrotron Radiation Facility (BSRF). For the X-ray absorption near edge structure (XANES) part, the experimental absorption coefficients as function of energies m (E) were processed by background subtraction and normalization procedures and reported as “normalized absorption” with E_0 = 7709 eV for all the

measured samples and Co foil standard. For the extended X-ray absorption fine structure part, the Fourier transformed (FT) data in *R* space were used for comparison. The Brunauer-Emmett-Teller (BET) specific surface area was determined by using N_2 sorption isotherm measurements at -196 ℃ on Micrometrics TriStar 3020 equipment. Temperature-programmed reduction (TPR) was carried out in a Micromeritics AutoChem II 2920 equipped with a thermal conductivity detector and a mass spectrometry detector. Typically, 50 mg of the catalyst was placed in a U-shaped quartz tube reactor and calcined at 100 °C for 1 h under helium atmosphere, then reactor was cooled down to 50 °C, followed by hearting the reactor to 1000 °C at a ramping rate of 10 °C/min with H_2/He with a total gas flow of 50 mL/min.

Electrochemical Measurements: All of the electrochemical measurements were conducted by using a CHI760E potentiostat in a typical three-electrode setup with O_2-saturated 1 M KOH solution. A Pt-foil as the counter electrode and Hg/HgO with 1 M KOH filling solution as the reference electrode. The as-measured potentials (versus Hg/HgO) were calibrated with respect to the RHE. A glassy carbon electrode with a diameter of 3 mm covered by a thin catalyst film was used as the working electrode. Typically, 5.6 mg catalyst and 2.4 mg acetylene black were suspended in 8 ml isopropanol-water solution with a volume ratio of 4:1 to form a homogeneous solution assisted by ultrasound for 3 h. Then 0.95 mL as prepared solution mixed with 50 μL Na^+ exchanged Nafion solution to form a homogeneous ink assisted by ultrasound for another 0.5 h. The Na^+ exchanged Nafion solution was prepared by mixing commercial Nafion® solution and 0.1 M NaOH solution with a volume ratio of 2 to 1[1]. Then, 2 μL of the ink was spread onto the surface of the glassy carbon electrode (mass loading: 0.028 mg cm^{-2}). The cyclic voltammetry (CV) measurements were performed between 0.9254 and 1.7254V_{RHE} (0 and 0.8 $V_{Hg/HgO}$) at 10 mV s^{-1}, the CV tests were carried out for three times, and the mean and standard deviation of the three test results were calculated. The potentials were corrected to compensate for the effect of solution resistance and calculated using the following equation: $E_{iR\ corrected}$ =

E - *i*R, where R is the uncompensated ohmic solution resistance measured via iR compensation module in CHI760E with comp level of 95%.

Alkaline water electrolyze measurements: The Alkaline water electrolyze (AWE) measurements were performed on a single-cell configuration with a reactive area of 1 cm^2. Commercial polyphenylene sulfide polymer film (UTP-220, thickness 0.22 mm, Agfa ZIRFON PERL, Belgium) and Raney Ni (John Cockerill, Suzhou, China) were used as diaphragm and cathode, respectively. The anode of AEW is a 100-mesh nickel mesh loaded with PBCO catalyst. The catalyst loading is 5 mg/cm^2. The performance of AWE was determined using 30% KOH solution as the electrolyte.

Differential electrochemical mass spectroscopy (DEMS) measurements: DEMS measurements were carried out to determine the ^{18}O-labeled OER reaction products of LSCO-0.5 catalysts during OER process using a QAS 100 device (Linglu Instruments, Shanghai). A saturated Ag/AgCl electrode and a Pt wire were used as reference electrode and counter electrode, respectively. The working electrodes were prepared by sputtering Au onto 50 μm thick porus PTFE films. Then, the catalysts were drop-cast onto the Au with a loading mass of 1 mg cm^{-2}. First, the catalysts were labeled with ^{18}O isotopes by performing 10 CV cycles at a scan rate of 10 mV/s in ^{18}O-labed 1 M KOH solution between 0.2 and 0.8 $V_{Ag/AgCl}$. Afterwards, ^{18}O-labeled electrodes were rinsed with ^{16}O water for five times to remove the remaining $H_2{}^{18}O$. Finally, the electrodes were carried out CV cycles in ^{16}O 1 M KOH solution at the above potential window and scan rate. At the meantime, gas products of different molecular weights generated during OER process were measured in real time by mass spectroscopy. Since catalysts were thoroughly rinsed with ^{16}O water after ^{18}O-labelling, it is unlikely that ^{18}O species adsorbed on the surface contribute substantially to the observed $^{34}O_2$ ($^{16}O^{18}O$) signals. Thus, it can be determined to investigate the participation of lattice oxygen from catalysts in OER by measuring the $^{34}O_2$ signals.

eQCM measurements: Electrochemical quartz crystal microbalance (eQCM) was performed in a 1 M KOH solution to detect the mass change for the PBCOs during

OER. An AT-cut quartz crystal with a fundamental frequency of 9.12 MHz $\pm$50 kHz. coated was used. The Au polycrystals coated with thin films of catalysts by spin coating deposition were used as working electrode. The working electrode was then mounted into a three-compartment electrochemical cell. A Hg/HgO electrode and a Pt wire were used as the reference electrode and counter electrode, respectively. The frequency change was measured by a Q-sensor analyser (QE 401) equipped with the QEC 401 electrochemistry module. Mass and dissipation were recorded along with the CV curves. All EQCM-D parts and sensors were purchased from Biolin Scientific.

Calculation Setup: First-principles calculation was carried out using VASP (Vienna *Ab-initio* Simulation Package)[2,3]. The projector augmented wave (PAW) pseudopotential with the PBE generalized gradient approximation (GGA) exchange correlation function was utilized in the computations[4]. The cutoff energy of the plane waves basis set was 500 eV and a Monkhorst-Pack mesh of $4\times4\times1$ was used in K - sampling[5]. All structures were spin polarized and all atoms were fully relaxed with the energy convergence tolerance of 10^{-4} eV per atom, and the final force on each atom was < 0.02 eV $Å^{-1}$.

Valence electron configuration for each element is that $5s^2 5p^6 6s^2$ in Ba, $5s^2 5p^6 6s^2 4f^1$ in Pr, $3d^8 4s^1$ in Co, $2s^2 2p^4$ in O[6]. In order to correct the significant self-interaction error inherent to the standard DFT in describing localized *d*-electrons with strong correlations, an on-site Hubbard term Ueff was added to the open-shell *d*-electrons, 3.06 eV for Co, 4.0 eV for Pr.

For PBCO, a 7-atomic layer slab with (001) plane Co-O exposed layer was constructed with over 15 Å vacuum layer. Although we do not know if the (001) plane is the dominating surface of PBC, the (001) surface with BO-transition metal ions (e.g., Co and Fe) has been widely used as the catalytically active and stable surface for simple perovskite in DFT calculations[7,8]. We believe this is also the case for the double-perovskite catalysts PBC and PBSCF[6]. Accordingly, a 7-atomic layer slab with (001) plane containing Co-O exposed to a vacuum layer (~15 Å thick) was used for the DFT calculations.

The equation to calculate Gibbs reaction energy is shown below[6]:

$\triangle G_r = \triangle E_{0K} + \triangle ZPE + \triangle H_{0K\rightarrow 298.15K} - T\triangle S_{0K\rightarrow 298.15K} \pm neU$

in which $\triangle E_{0K}$ is the reaction energy at 0 K, $\triangle$ZPE is the reaction energy from zero-point vibration, $\triangle H_{OK\rightarrow 298.15K}$ and $\triangle S_{OK\rightarrow 298.15K}$ are enthalpy and entropy contribution from 0 K to 298.15 K for reaction energy, neU is the electrochemical potential energy of n electrons (system reduction +, oxidation -) under electrode potential U. The PBE functional predicts an over-binded triplet oxygen dimer, so its energy at 298.15 K was calculated as[6]:

$\triangle G_{O2} = 2G_{H2O} - 2G_{H2} + 4\times 1.23$ (eV)

The p or d band centnjer Ep(d) was calculated based on the following equation: Ep(d) = ∫ ρE dE/∫ ρ dE, where ρ is the projected electron DOS at the energy level of E[9].

For the pressure calculation, we set the PSTRESS to 0.001 and 40KB respectively to simulate the pressure applied during the mechanochemical process[8,9].

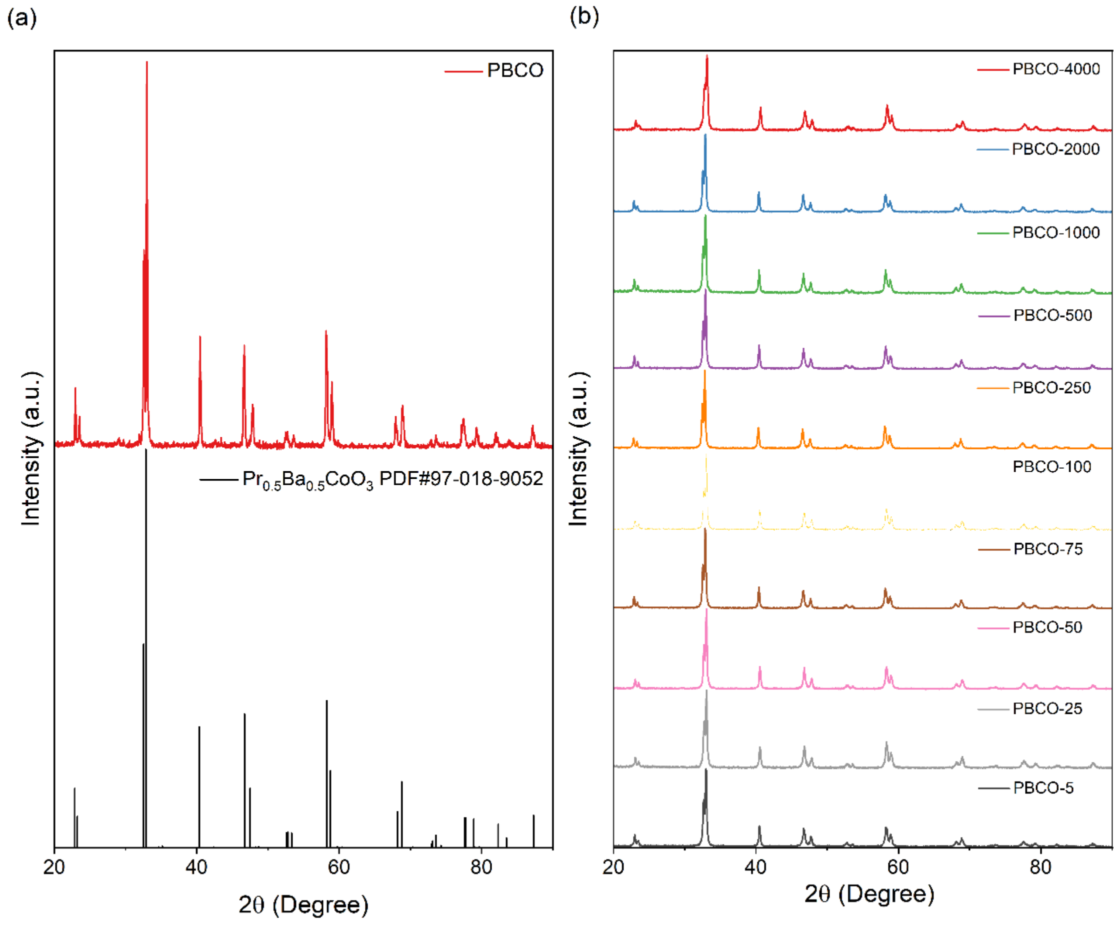

Figure S1. The XRD patterns of original PBCO without ball-milling treatment and all PBCO samples after ball-milling.

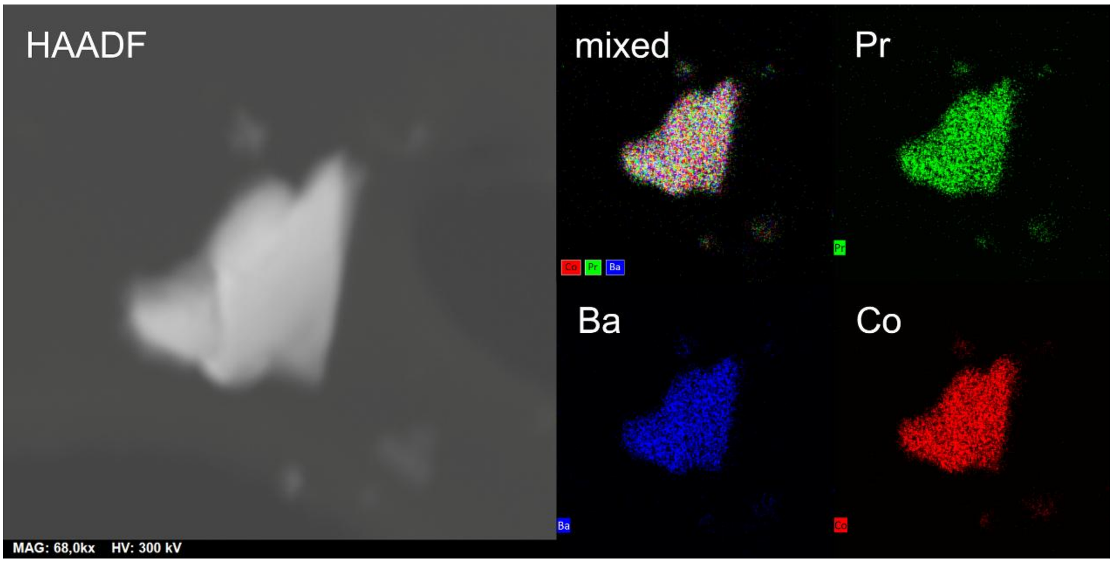

Figure S2. The EDS element mapping graphs of PBCO.

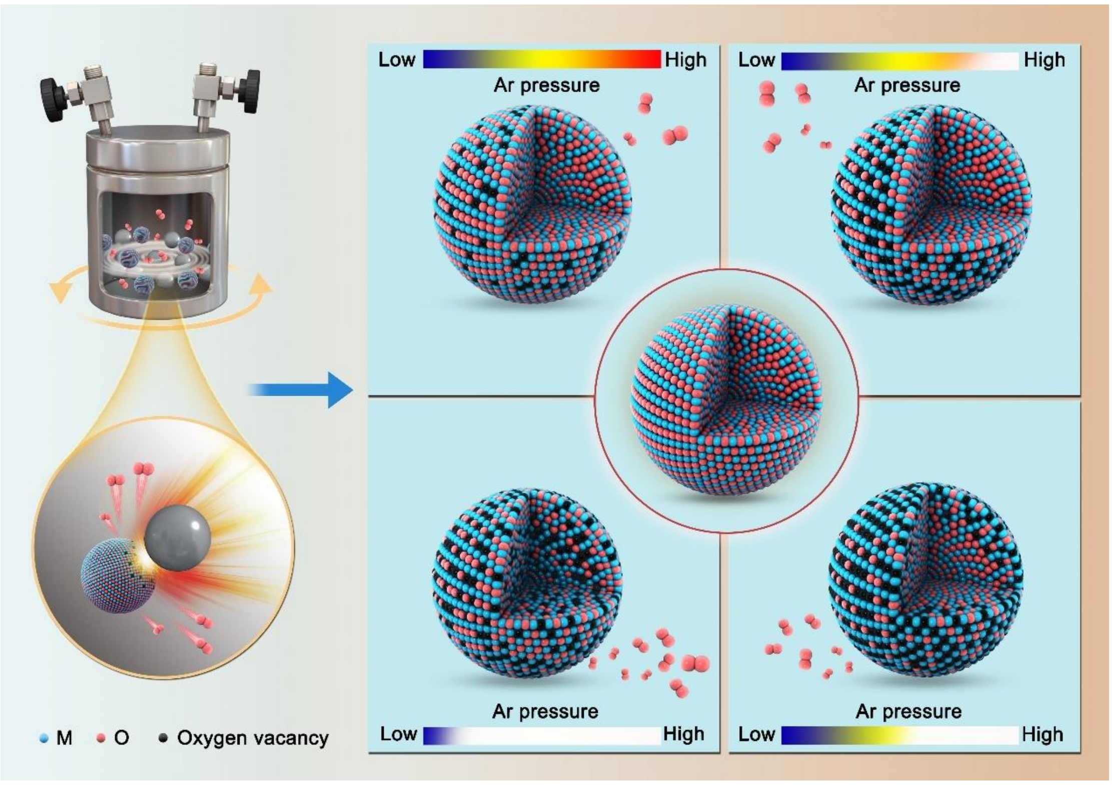

Figure S3. Schematic illustration for the mechanochemical synthesis process and spatial distribution regulation of V_O in PBCO. The two connected red balls in the figure represent oxygen molecules.

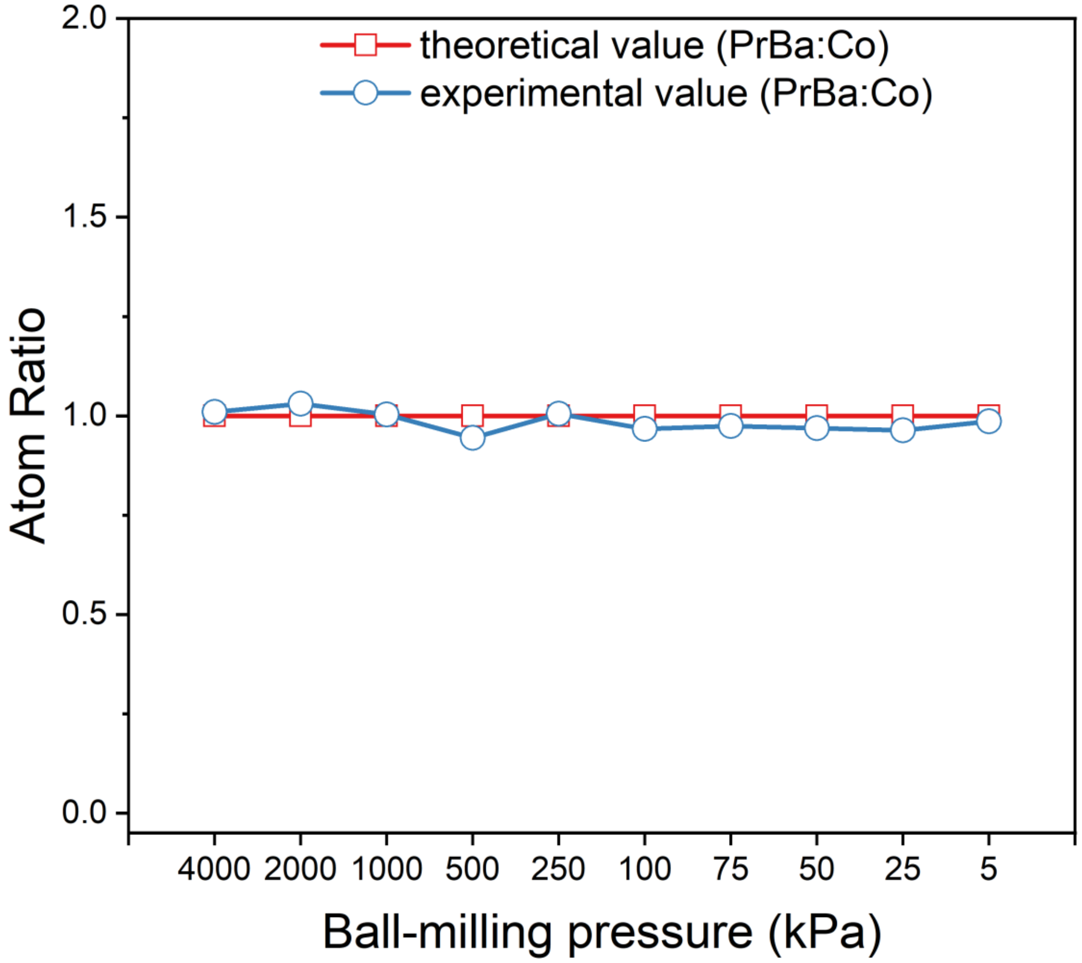

Figure S4. The relative metal ratio of as synthesis PBCO samples from ICP tests.

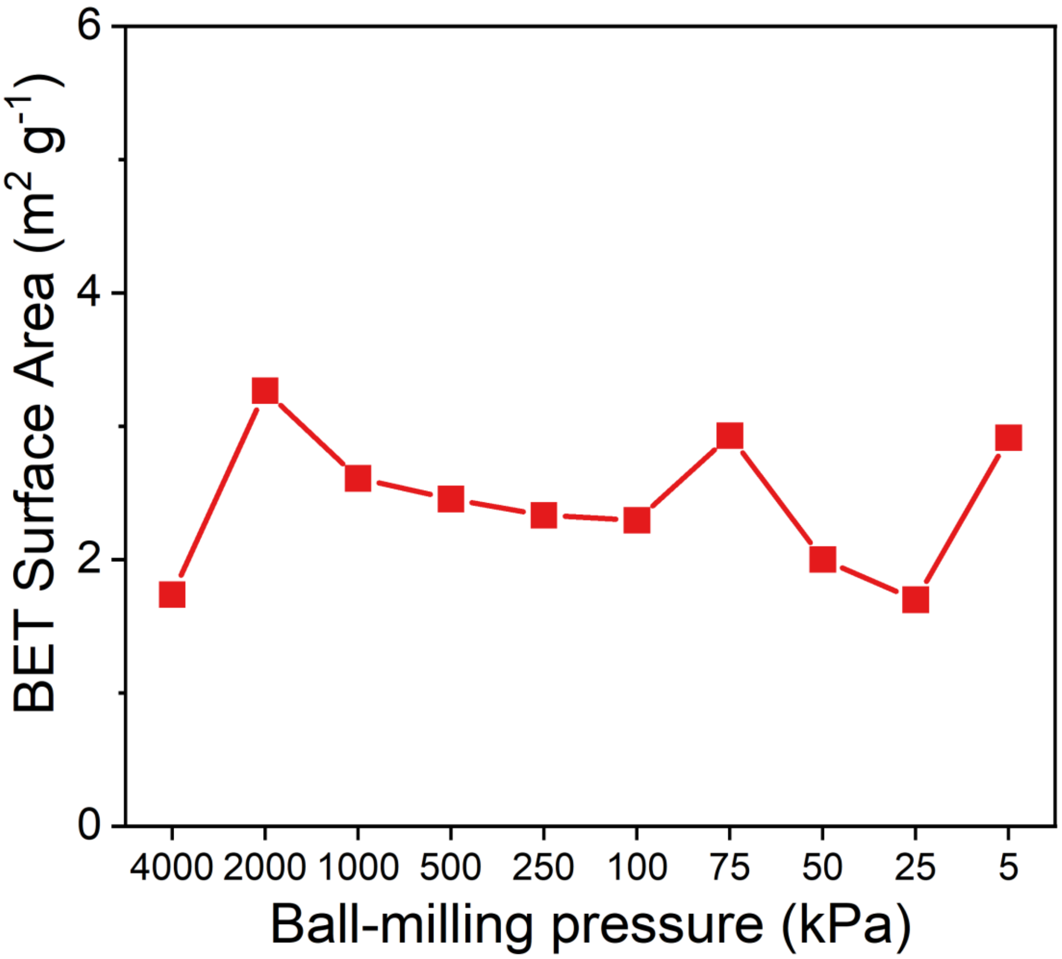

Figure S5. The BET specific surface area of PBCOs after ball-milling.

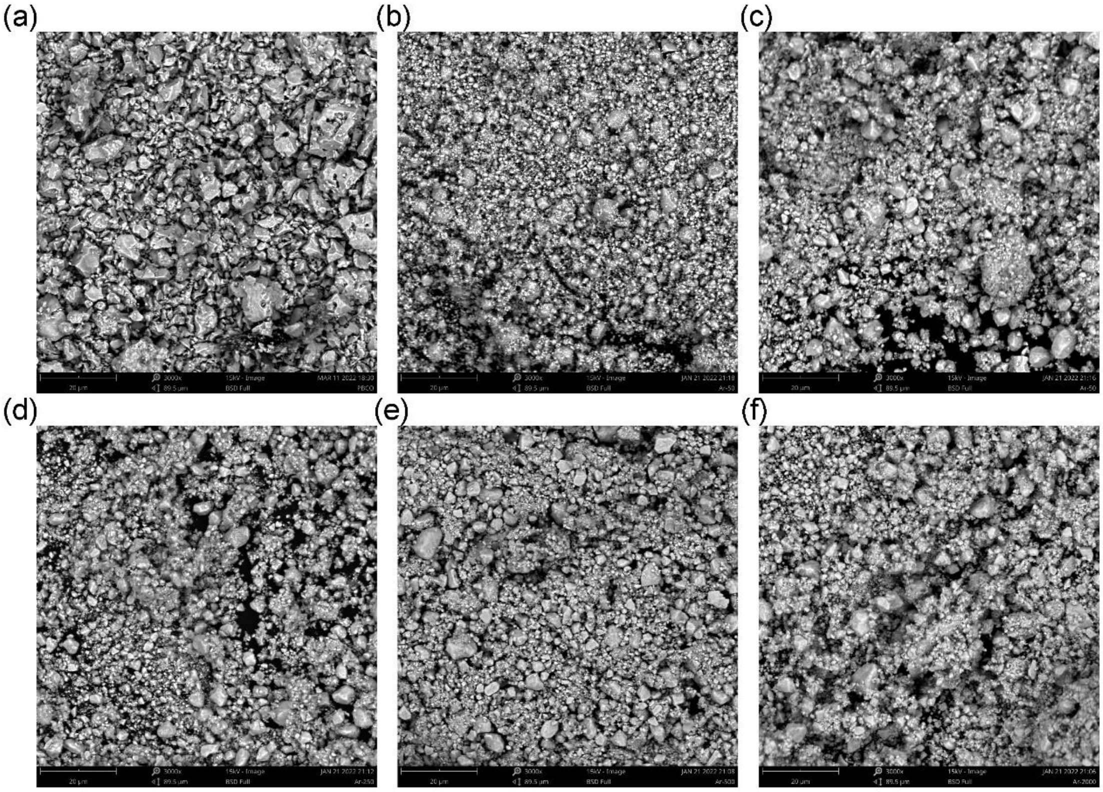

Figure S6. The SEM images of (**a**) PBCO, (**b**) PBCO-5, (**c**) PBCO-50, (**d**) PBCO-100, (**e**) PBCO-500, (**f**) PBCO-2000.

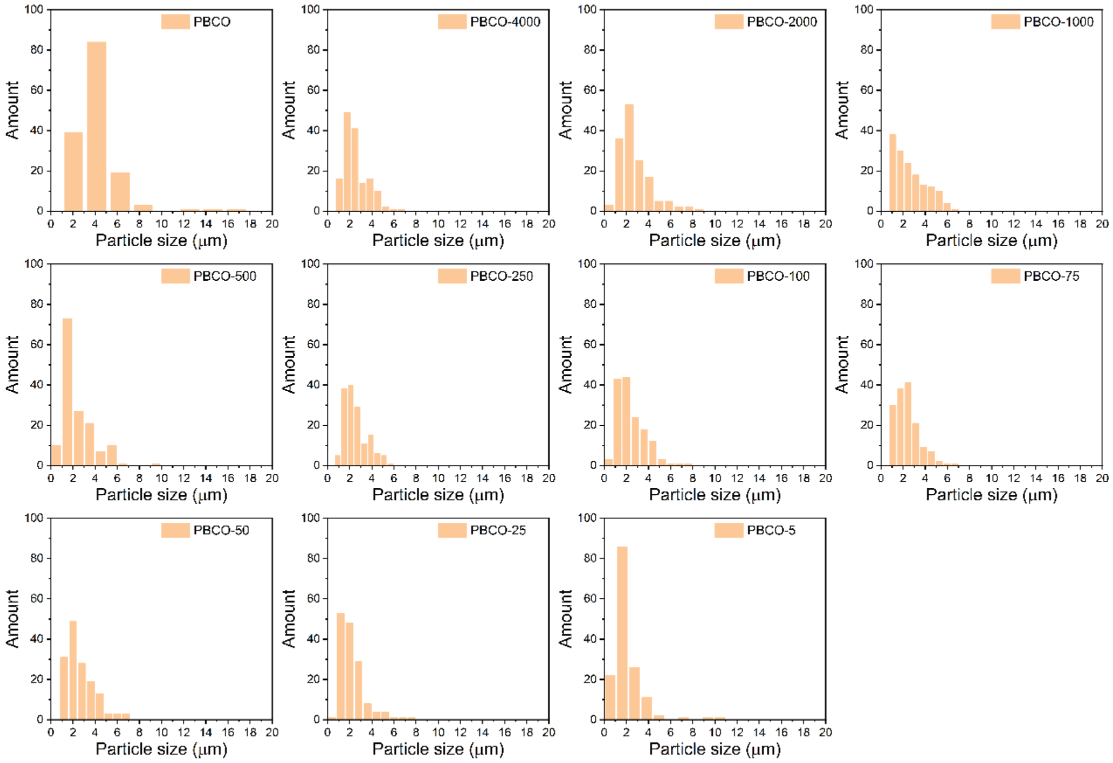

Figure S7. The particle size distribution graphs of all PBCO samples. The data are derived from 150 randomly selected particles in the SEM images.

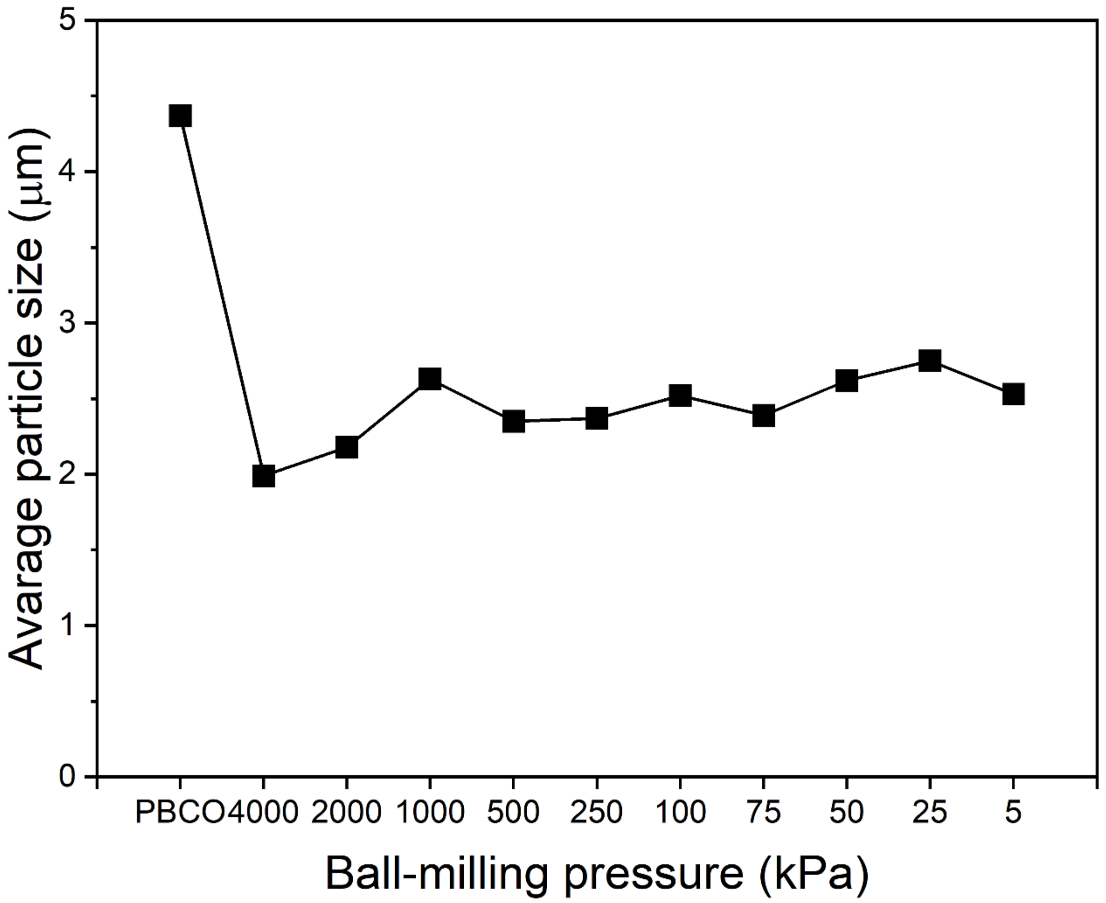

Figure S8. The average particle size of all PBCO samples.

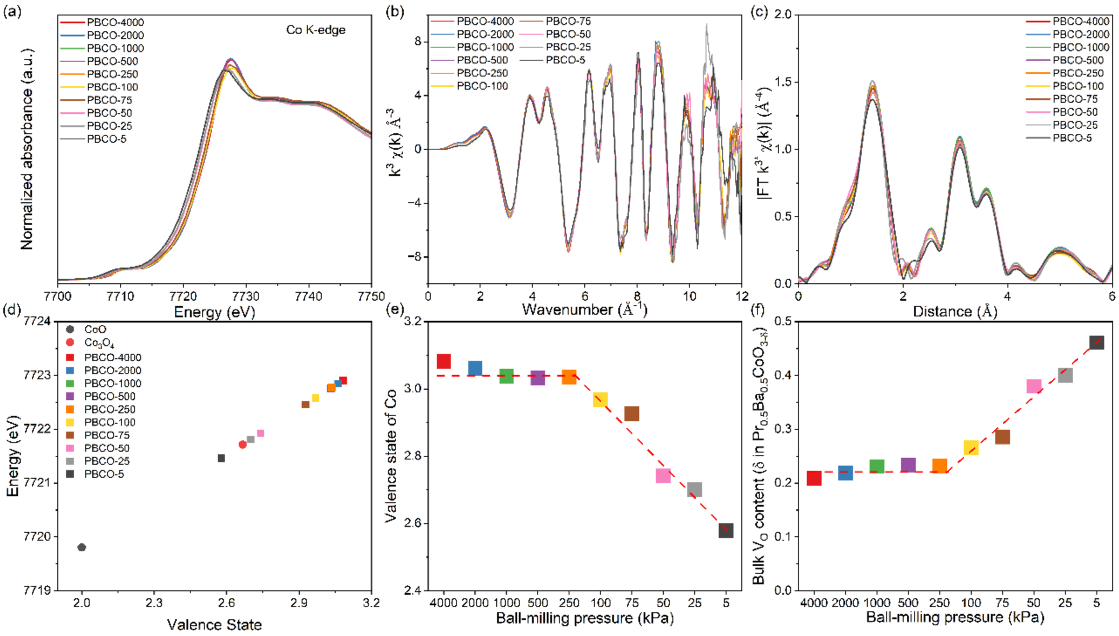

Figure S9. (a-c) Co K-edge XANES, EXAFS k3χ(k) Fourier transform (FT) and Co K-edge EXAFS spectra of PBCOs. **(d-f)** Line fit profile of valence state of Co, calculated valence state of Co and calculated oxygen defects content of PBCOs.

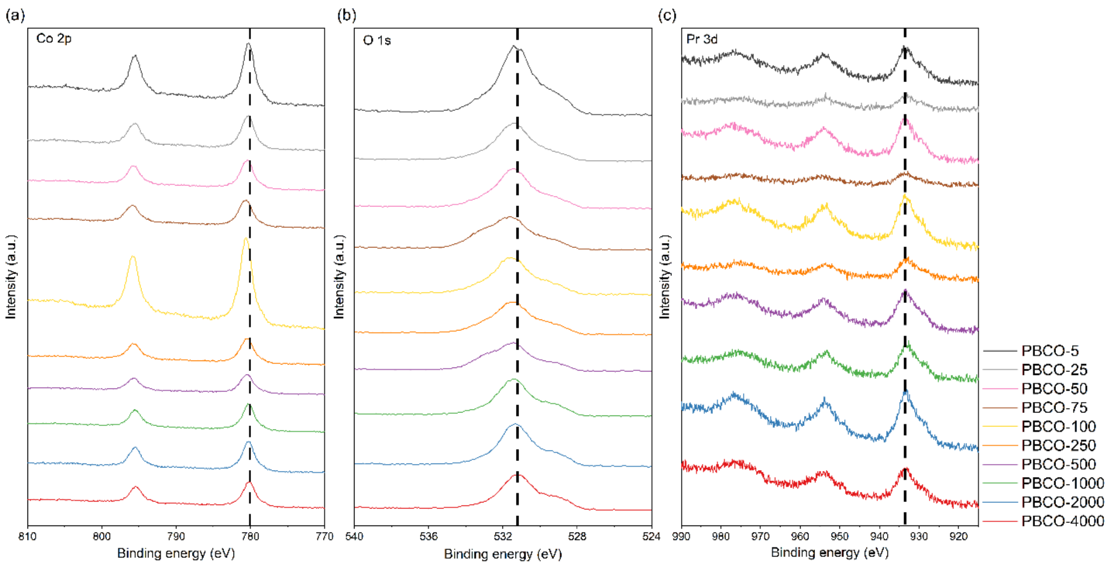

Figure S10. (**a**) The Co 2p XPS spectrum of PBCOs, (**b**) The O 1s XPS spectrum of PBCOs, (**c**) The Pr 3d XPS spectrum of PBCOs.

Although Pr has the possibility of existing in +3 and +4 valence states, in the perovskite oxide system, Pr generally exists in the valence state of +3. We conducted XPS characterization of the Pr elements in all samples. The results (Figure S13c) showed that the peak positions and shapes of Pr 3d XPS in all samples were almost unchanged, especially with the peak positions remaining consistent. This proves that the electronic cloud density, valence state, and other electronic structures of Pr on the material surface were not affected by the concentration and distribution of V_O. Therefore, when we quantitatively calculated the bulk V_O concentration of the material, we fixed the valence of Pr as +3 and the valence of Ba as +2. Through XAFS quantitative characterization of the valence state of Co and combined with the composition of metal elements in the sample. We obtained the total positive charge and total negative charge number, and then obtained the stoichiometric number of oxygen, as well as the content and concentration of V_O.

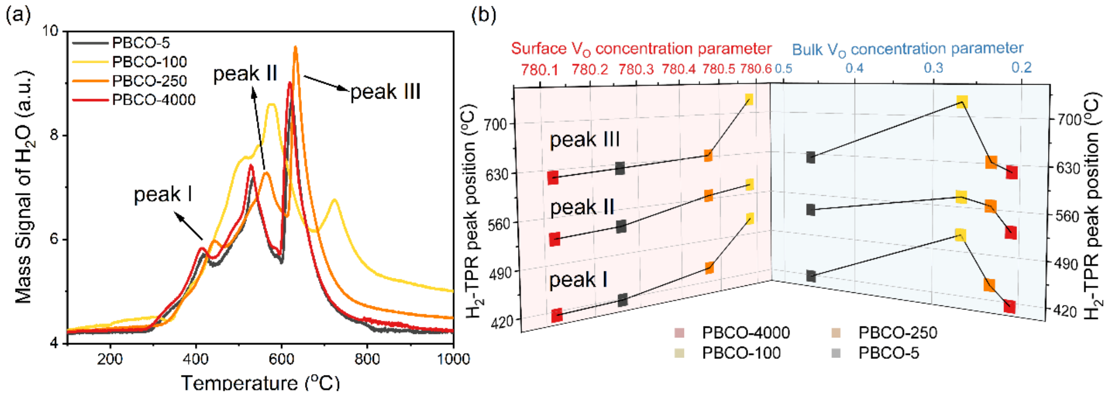

Figure S11. (a) The H_2-TPR-MS pattern of PBCO-4000, PBCO-250, PBCO-100, and PBCO-5. **(b)** The position of reduction peak in H_2-TPR-MS and its relationship to the surface/bulk V_O concentration parameters of PBCO-4000, PBCO-250, PBCO-100, and PBCO-5.

Then the H_2-temperature programmed reduction-mass spectrum (H_2-TPR-MS) was further performed on four different PBCO samples to further investigate the reducibility and surface V_O concentration of materials (Figure S9a). The first peak at 400-520 °C was due to the reduction of O^{2-} nearby Co^{3+} ($Co^{3+}\rightarrow Co^{2+}$) in the surface, the second peak at 520-580 °C was due to the reduction of O^{2-} nearby Co^{2+} ($Co^{2+}\rightarrow Co^{0}$) in the surface, the third peak at 600-730 °C was due to the reduction of O^{2-} in the subsurface. The position of three typical TPR peaks (peak I, II, and III) all show a volcano typed change trend from PBCO-4000 to PBCO-5 (Figure S9b). Because the higher the reduction temperature means that the material is more difficult to reduce, as well as the higher intrinsic surface V_O concentration. Therefore, the surface V_O concentration of materials also has a volcano-typed variation trend. Besides, the H_2-TPR spectrum and peak position of PBCO-4000 and PBCO-5 are similar, which show that PBCO-4000 and PBCO-5 has similar surface and near-surface V_O structure.

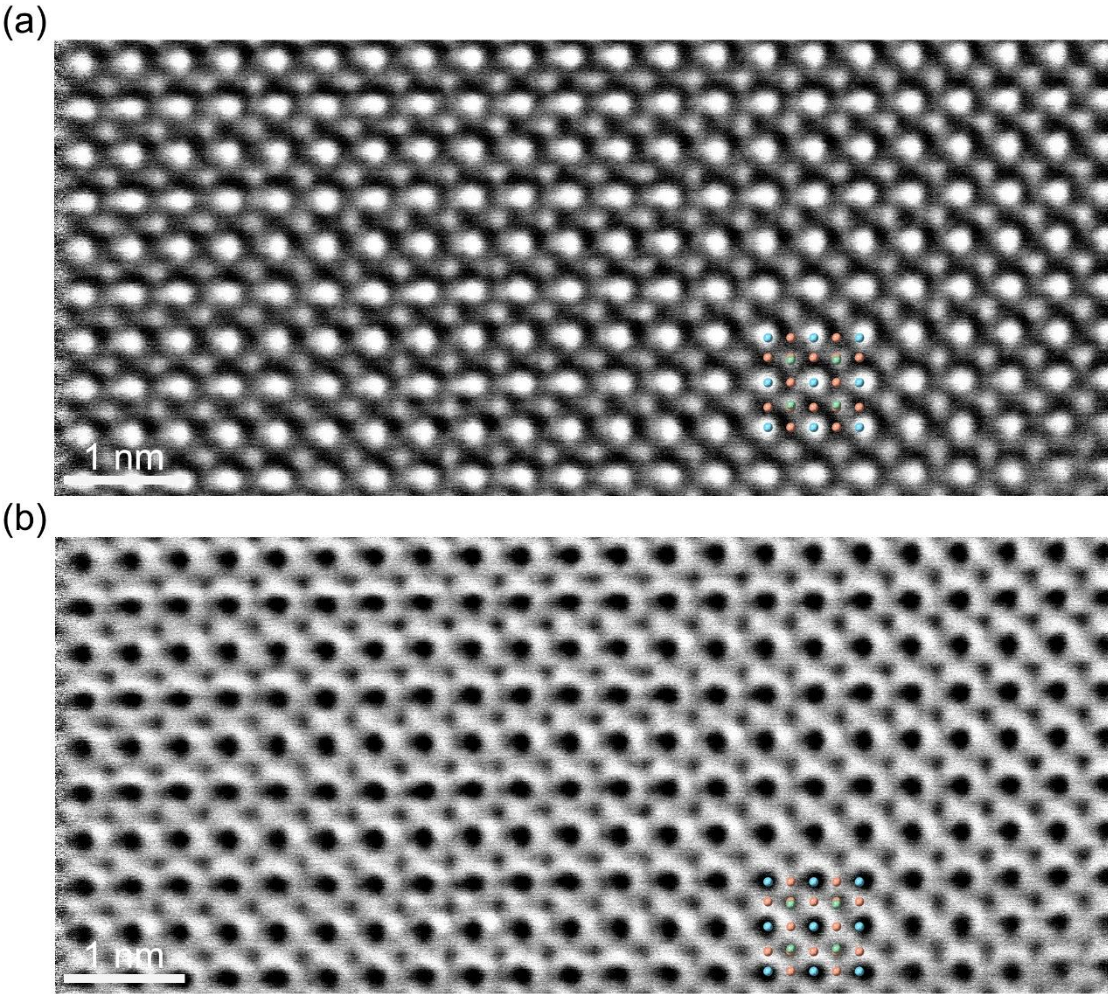

Figure S12. (**a**) The inverted ABF-STEM image of PBCO-100. (**b**) The original ABF-STEM image of PBCO-100. Inset is corresponding crystal structure of PBCO. The blue balls are Pr/Ba atoms, the green balls are Co atoms, the red balls are O atoms.

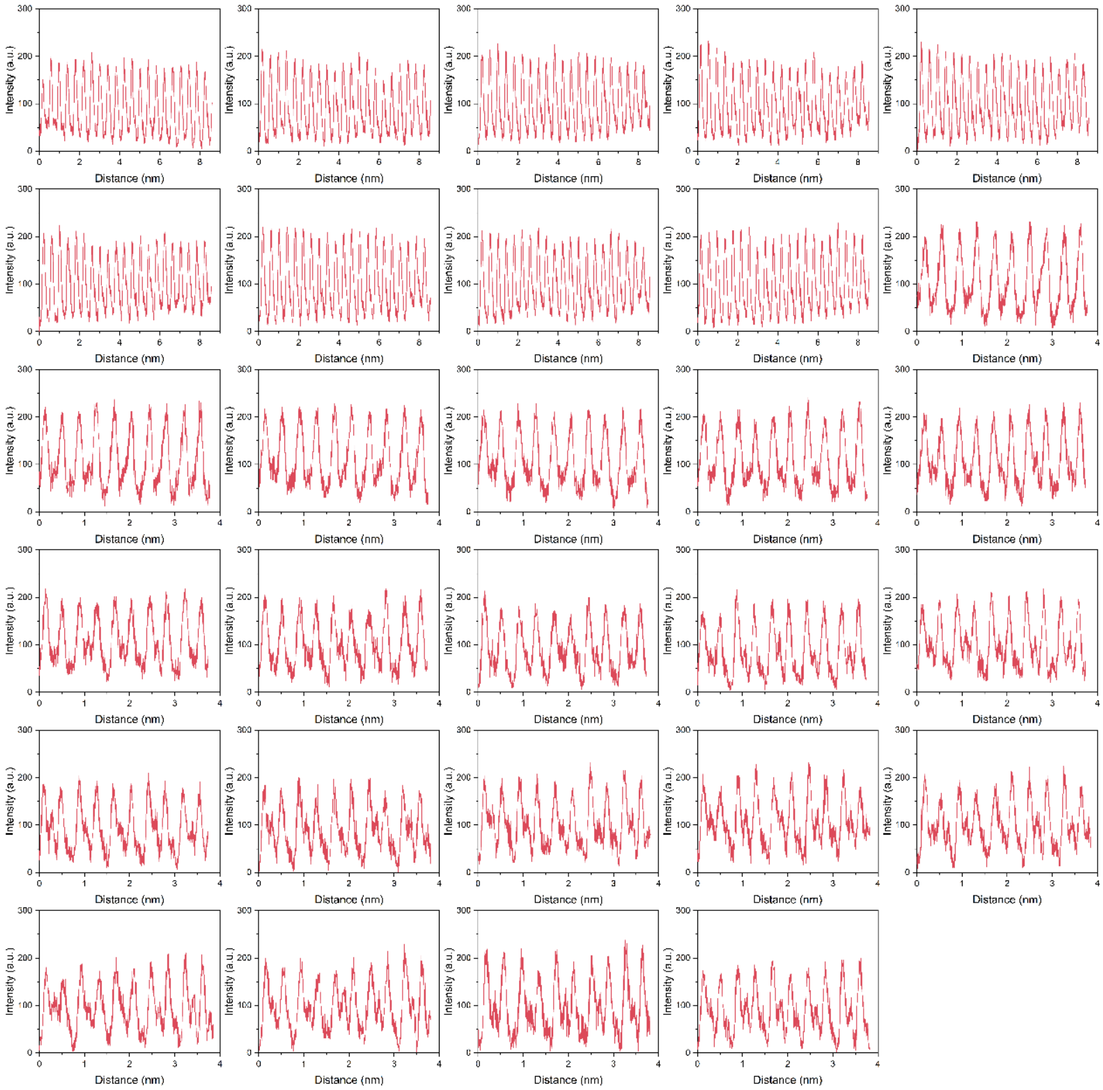

Figure S13. The intensity and relative intensity profiles of each O atoms column in Fig. S10.

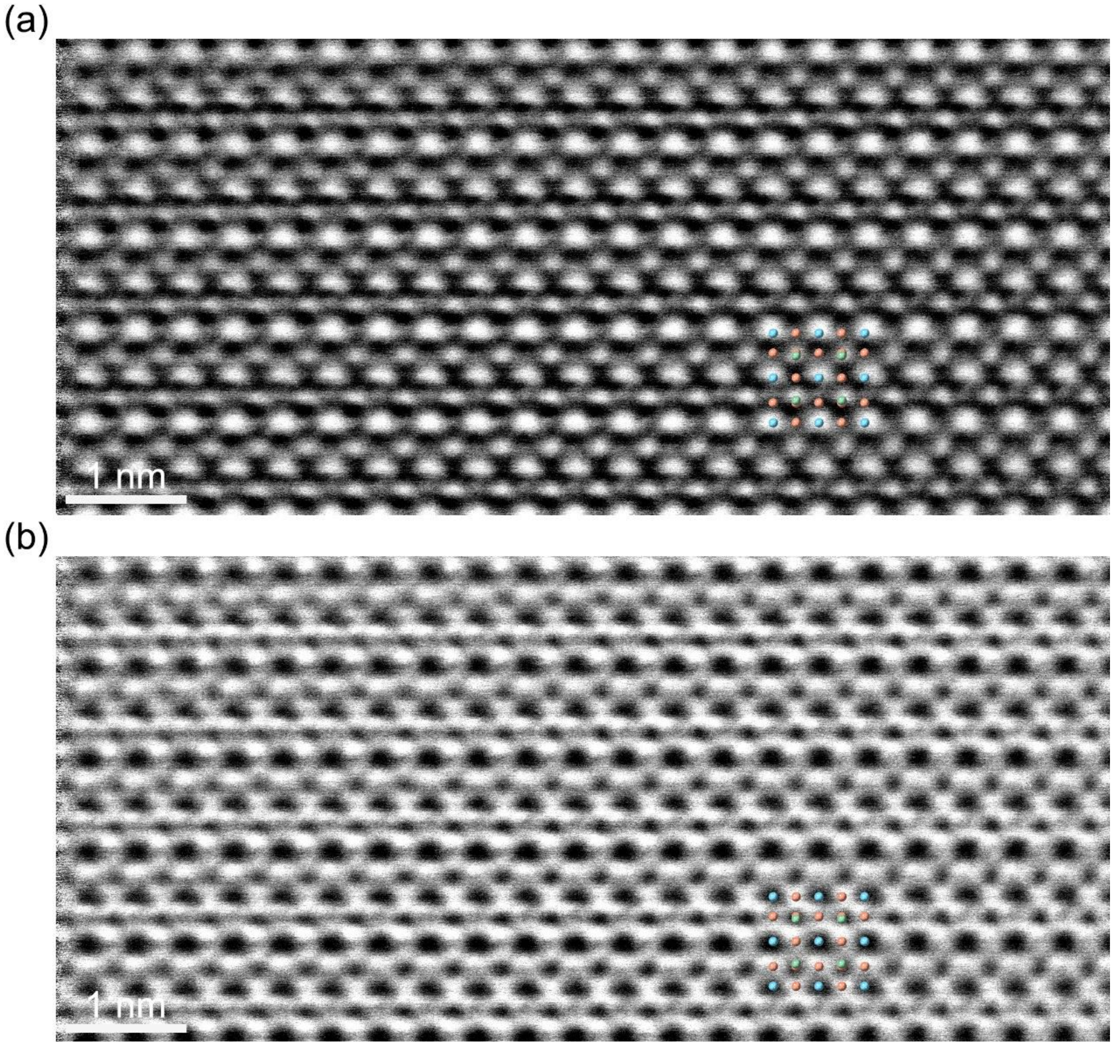

Figure S14. (**a**) The inverted ABF-STEM image of PBCO-4000. (**b**) The original ABF-STEM image of PBCO-4000. Inset is corresponding crystal structure of PBCO. The blue balls are Pr/Ba atoms, the green balls are Co atoms, the red balls are O atoms.

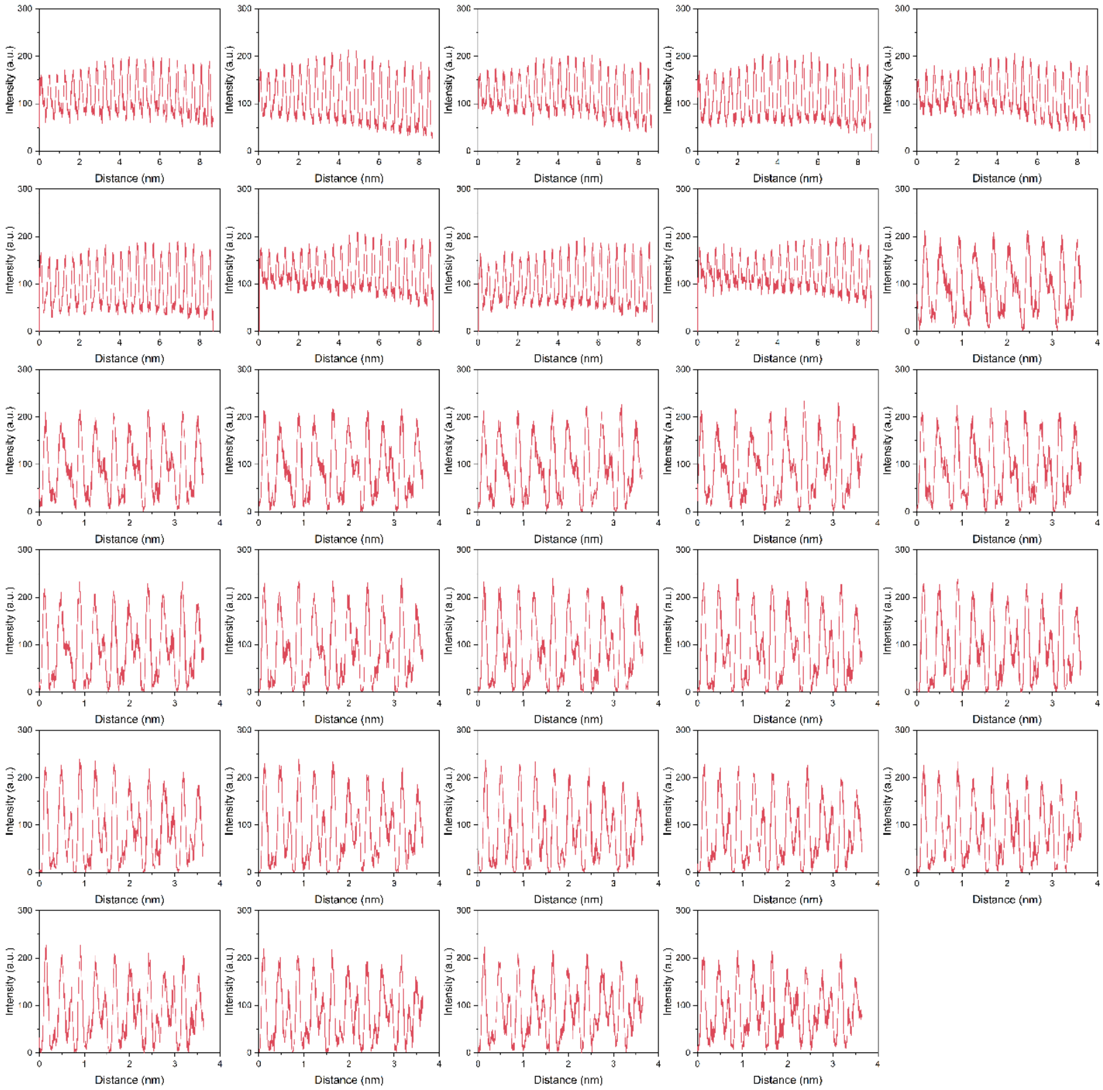

Figure S15. The intensity and relative intensity profiles of each O atoms column in Fig. S12.

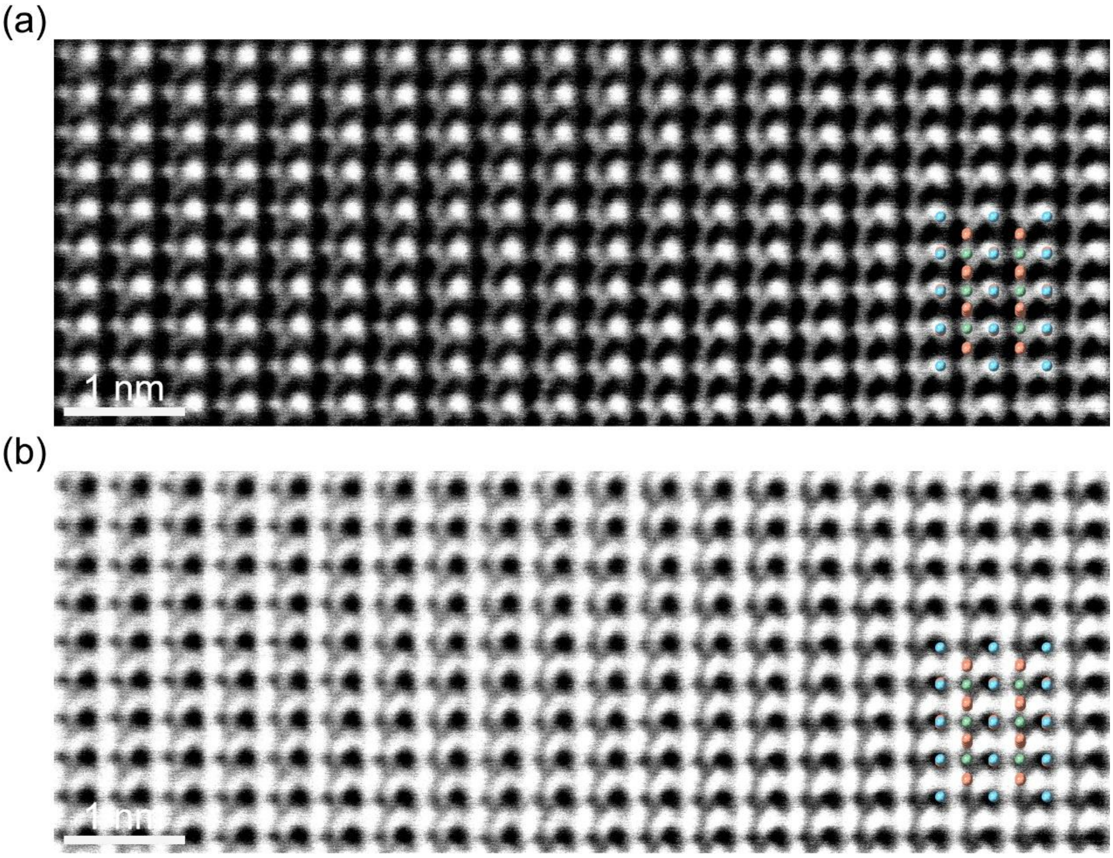

Figure S16. (**a**) The inverted ABF-STEM image of PBCO-5. (**b**) The original ABF-STEM image of PBCO-5. Inset is corresponding crystal structure of PBCO. The blue balls are Pr/Ba atoms, the green balls are Co atoms, the red balls are O atoms.

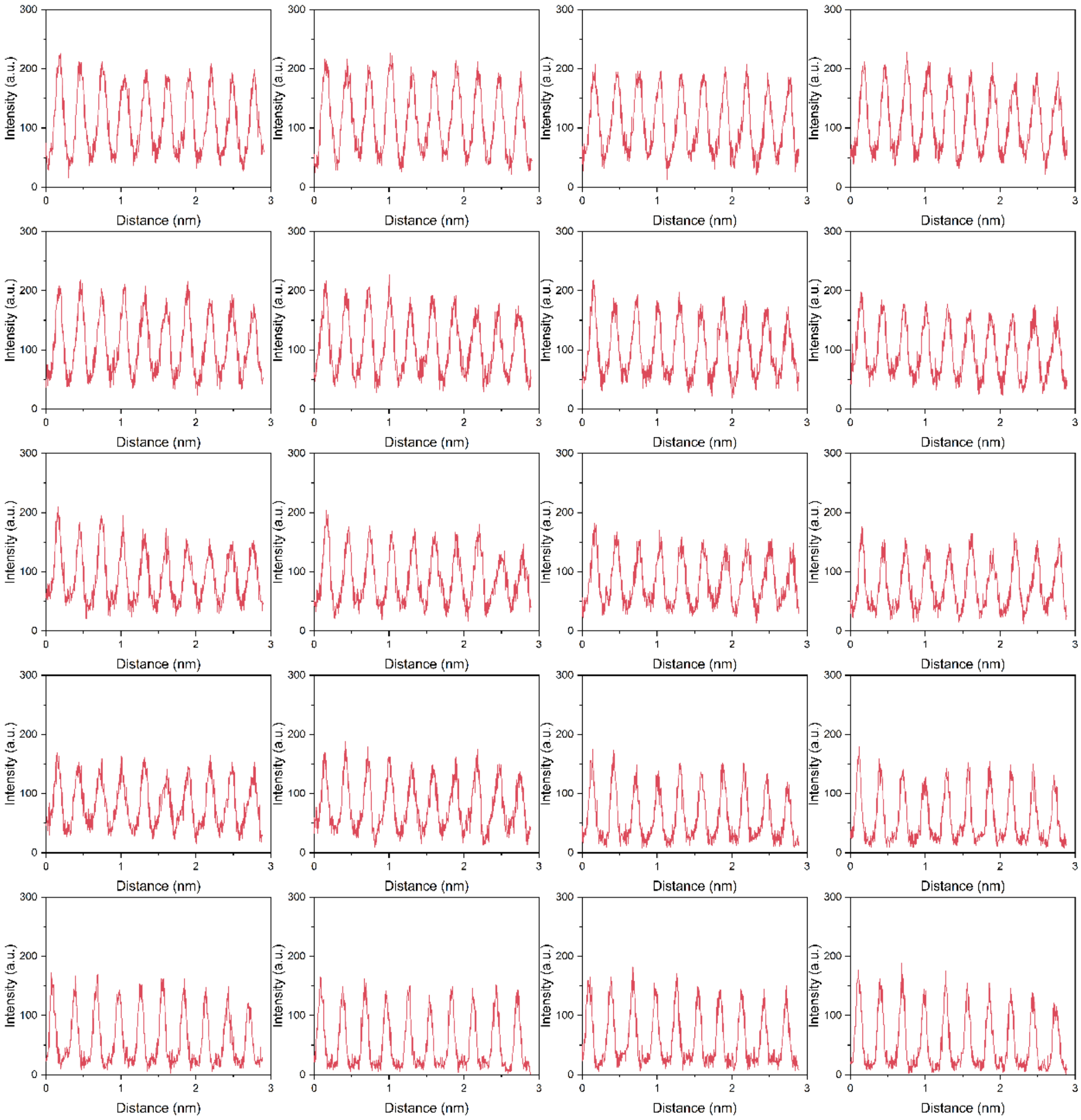

Figure S17. The intensity and relative intensity profiles of each O atoms column in Fig. S14.

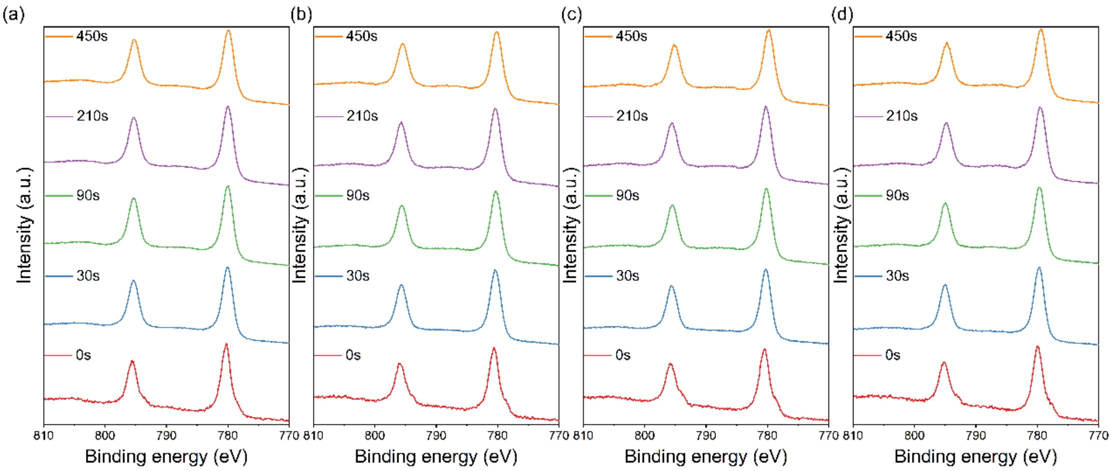

Figure S18. The Co 2p XPS in-depth profile after Ar plasma etching at different time of (**a**) PBCO-5, (**b**) PBCO-100, (**c**) PBCO-250, (**d**) PBCO-4000.

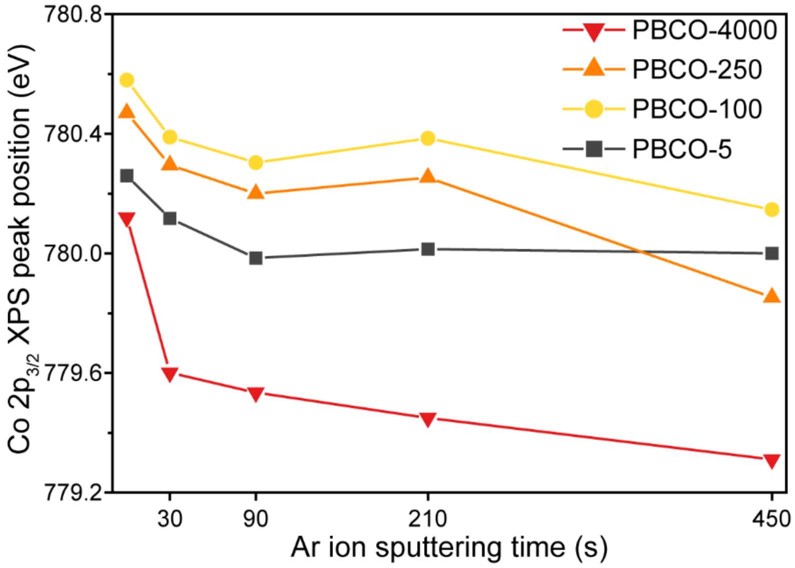

Figure S19. The position of Co $2p_{3/2}$ XPS peak on the surface of the PBCOs after Ar plasma etching at different time.

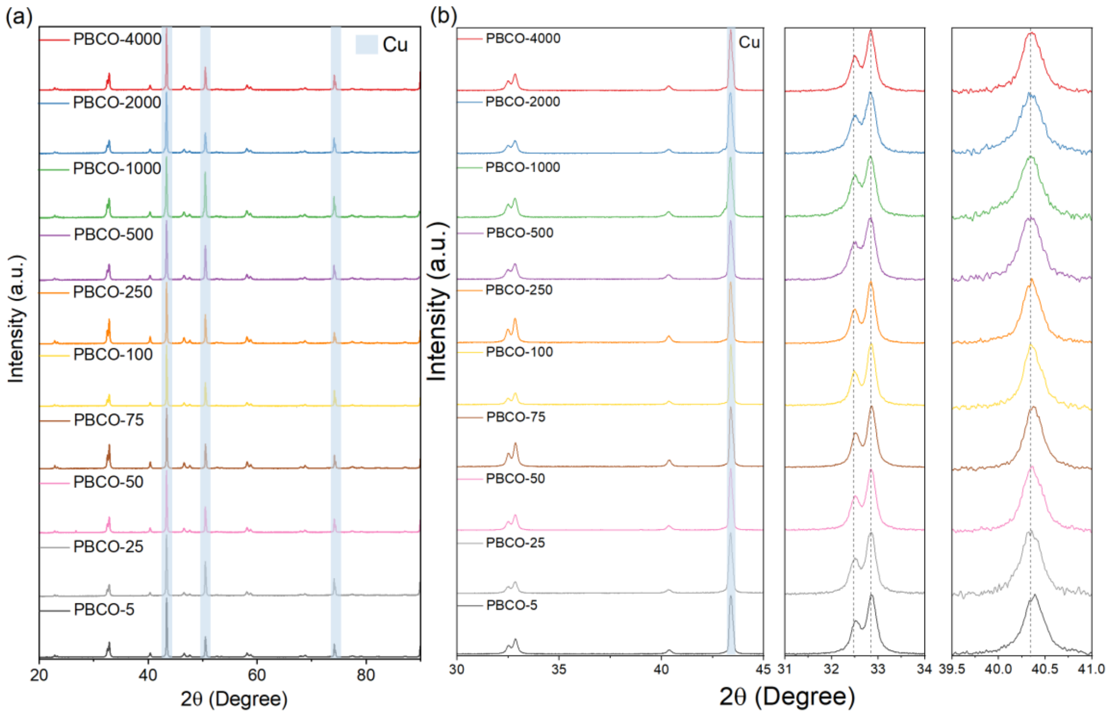

Figure S20. (a) The XRD patterns of PBCOs corrected by internal standard (Cu powders), **(b)** enlarged XRD patterns area of (**a**).

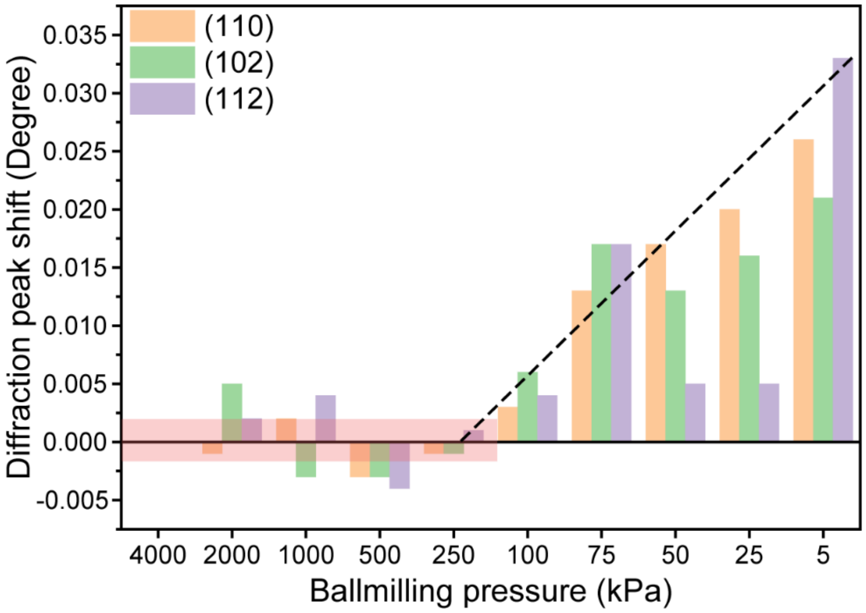

Figure S21. The peak position shift values of the three typical diffraction peaks ((110), (102), and (112)) relative to the PBCO-4000 in all PBCO samples (corrected by internal standard).

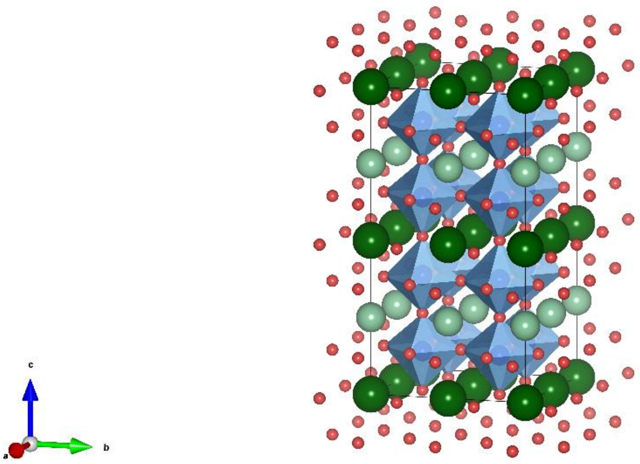

Figure S22. Crystalline structure model of PBCO supercell.

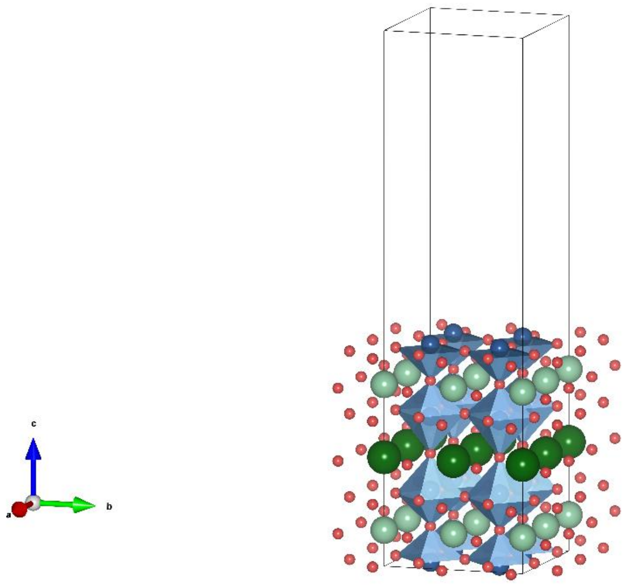

Figure S23. Crystalline structure model of PBCO (001).

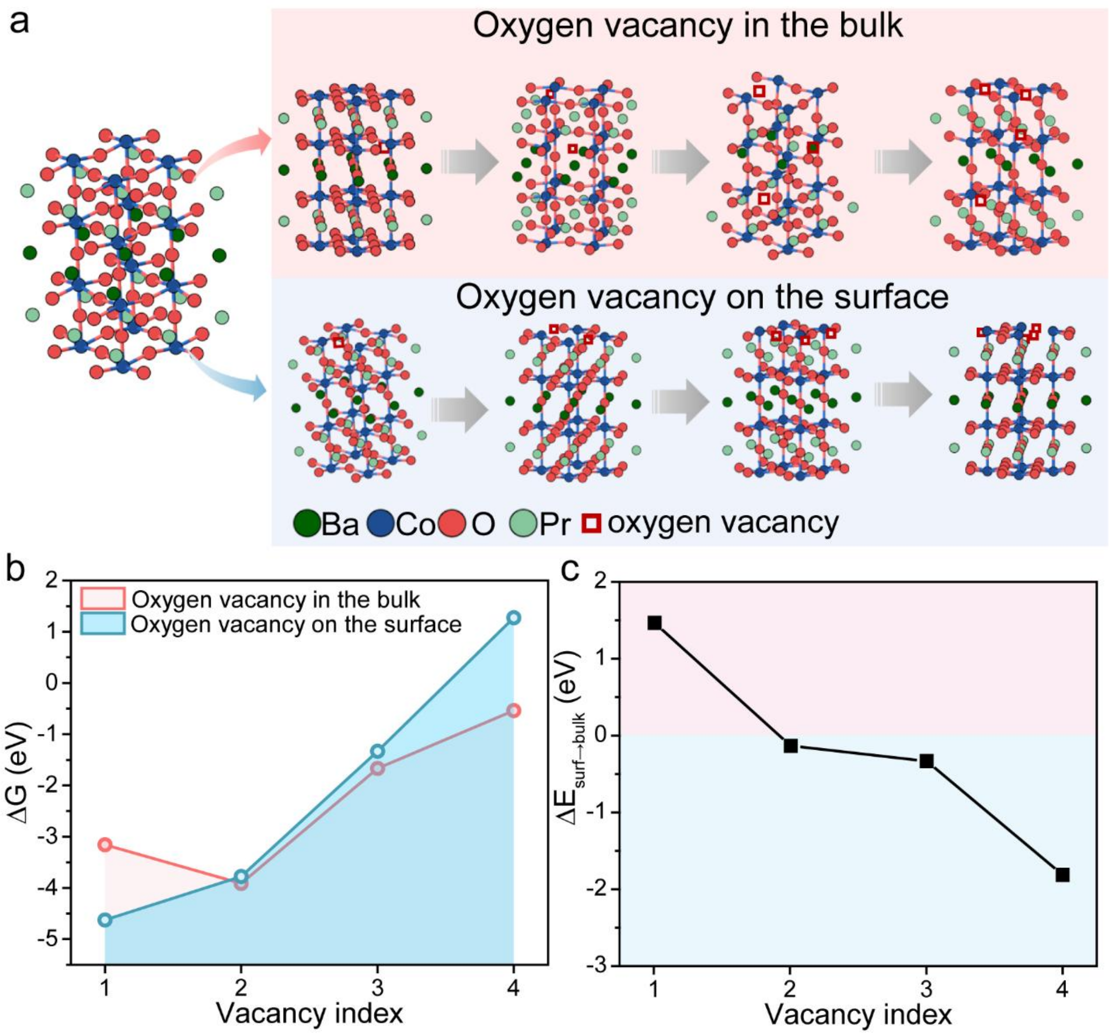

Figure S24. (a) Models for calculation of E_{vfb} and E_{vfs}. **(b)** The corresponding values of ΔG_{vfb} and ΔG_{vfs} for PBCO with different amounts of oxygen vacancies on different regions. **(c)** The corresponding values of V_O migration energy obtained by energy difference between ΔG_{vfb} and ΔG_{vfs} for PBCO with different amounts of oxygen vacancies.

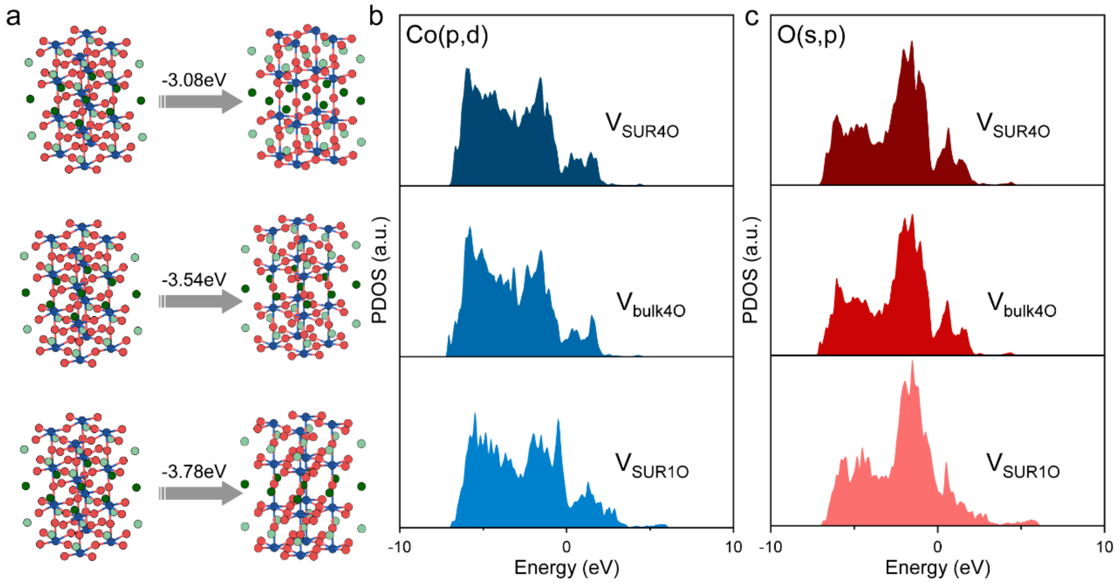

Figure S25. (a) ΔG_{vfs} for PBCO with oxygen vacancies pair on the meta-position, ortho-position and opposite-position, respectively. **(b-c)** PDOS of Co-*p*,*d*. (**b**) and O-*s*,*p*. (**c**) in the PBCO model with varied V_O concentrations.

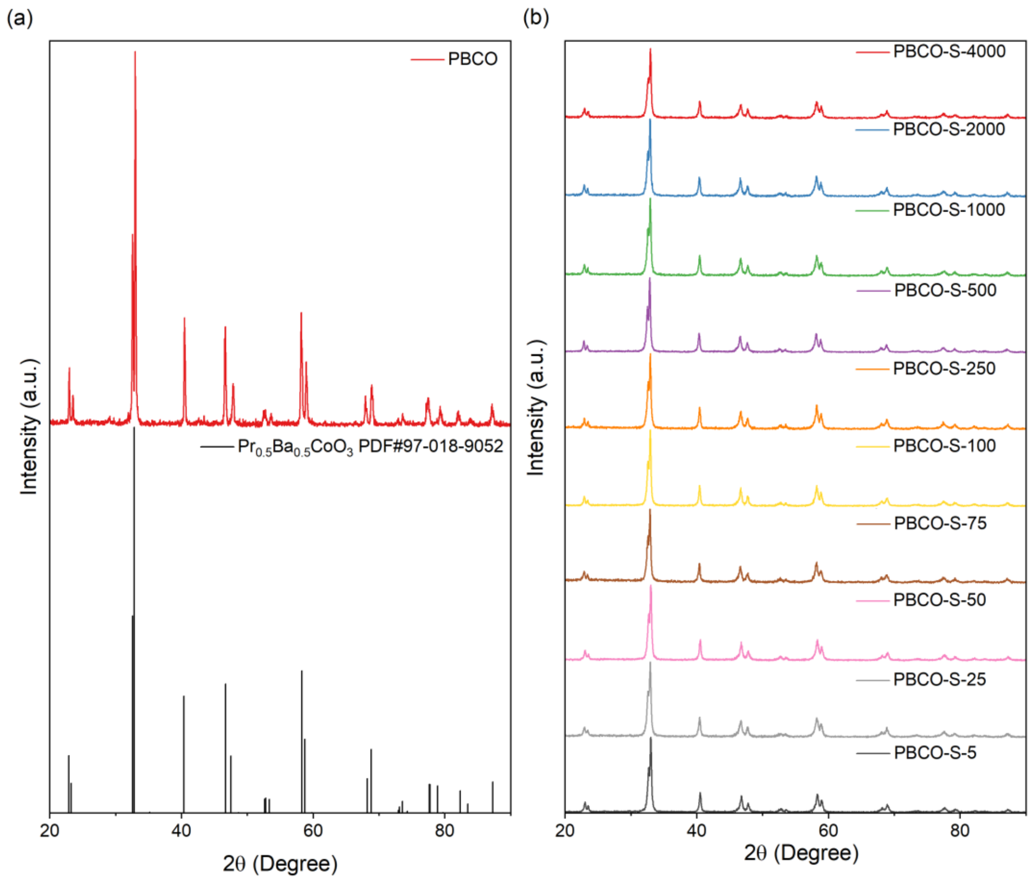

Figure S26. (a) The XRD results of original PBCO, (b) The XRD results of PBCO-S-4000 to PBCO-S-5.

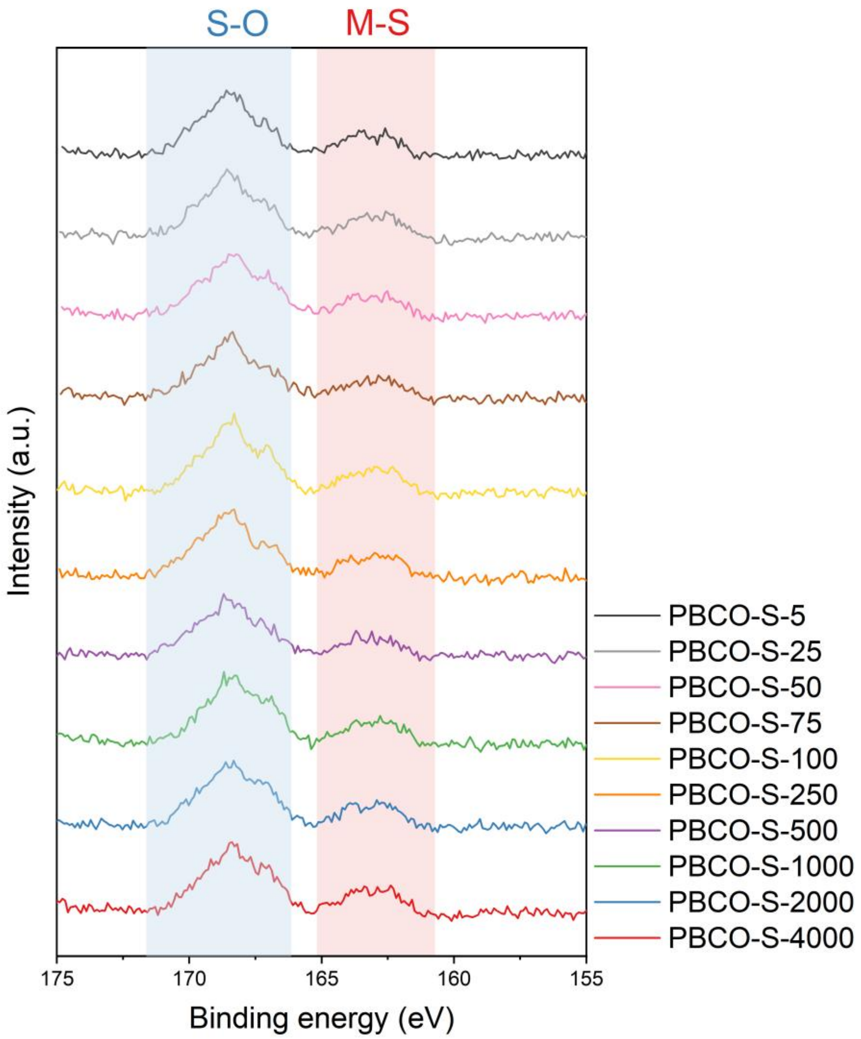

Figure S27. The S 2*p* XPS spectra of all PBCO-S samples.

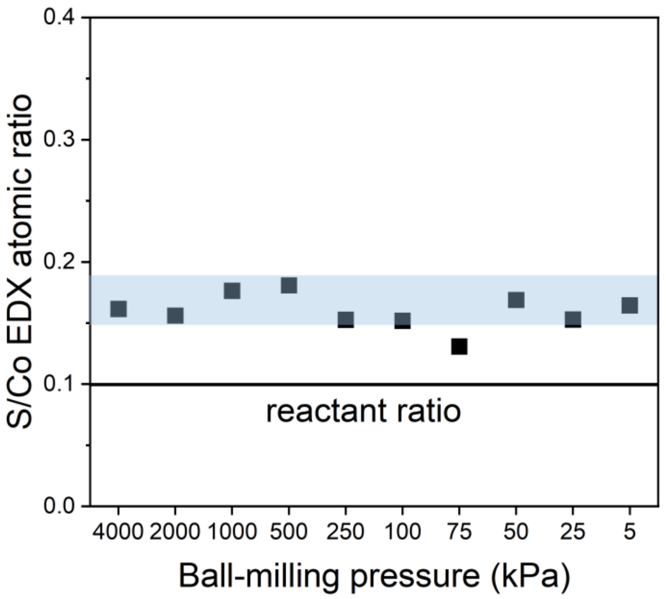

Figure S28. The S/Co atomic ratio of all PBCO-S samples from SEM-EDX.

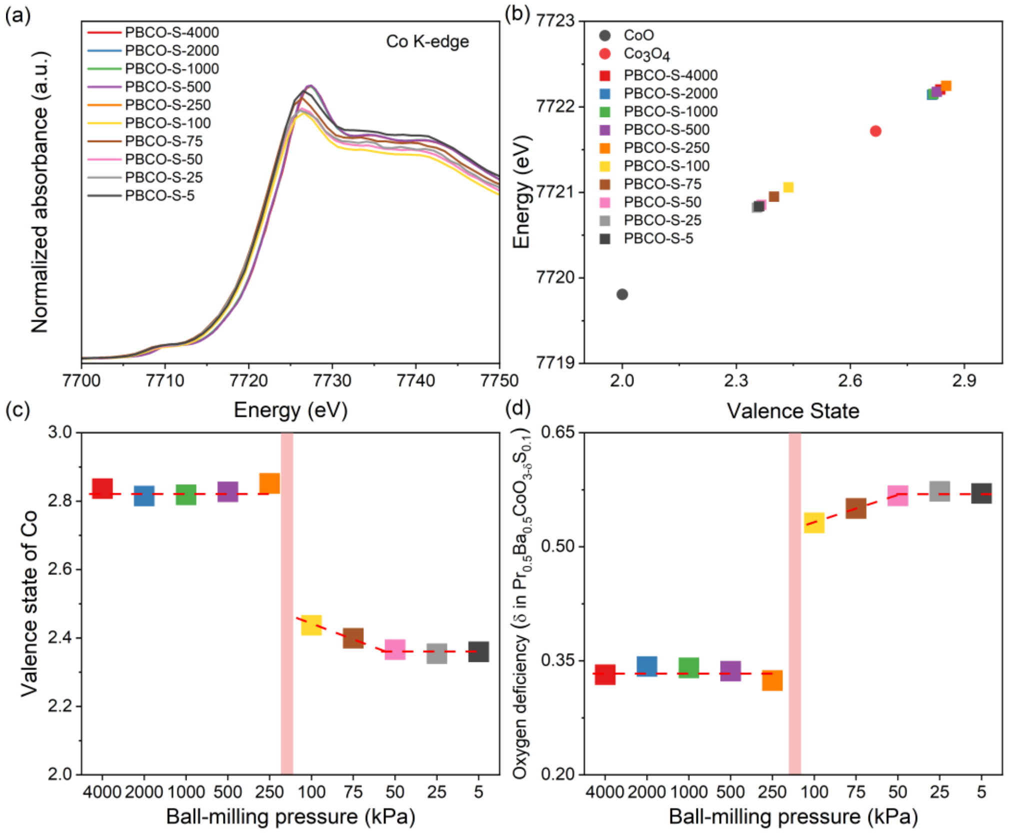

Figure S29. (a) The Co K-edge XANES spectra of all PBCO-S samples. (b) Line fit profile of valence state of Co. (c) Calculated Co valence state of all PBCO-S samples. (d) Calculated oxygen vacancies content of all PBCO-S samples.

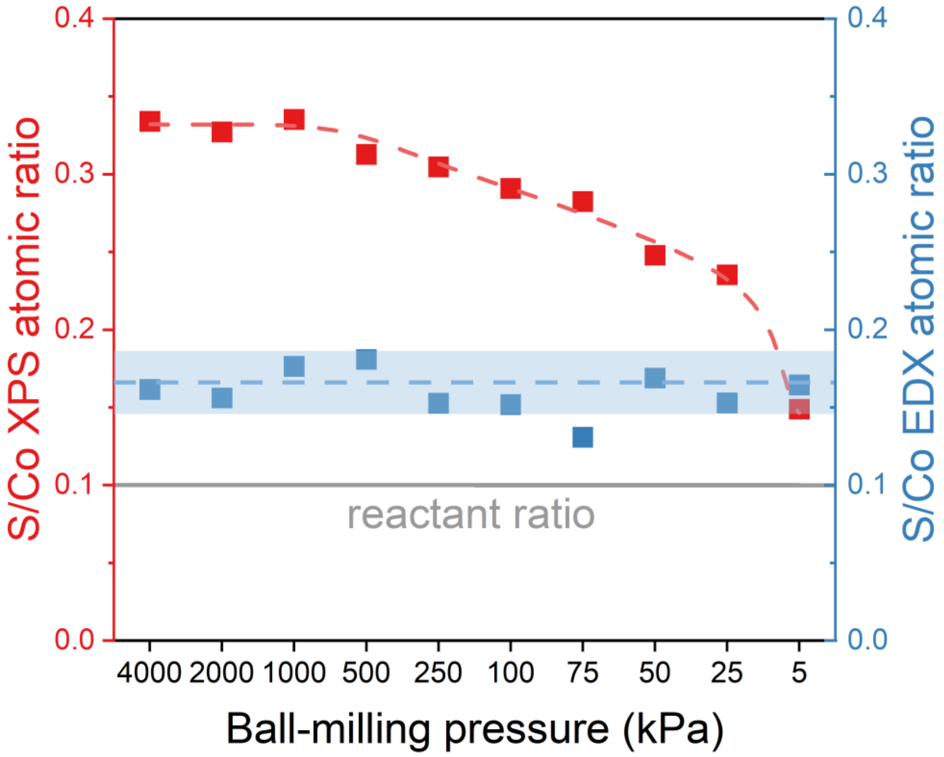

Figure S30. The S/Co atomic ratio of all PBCO-S samples from SEM-EDX and XPS.

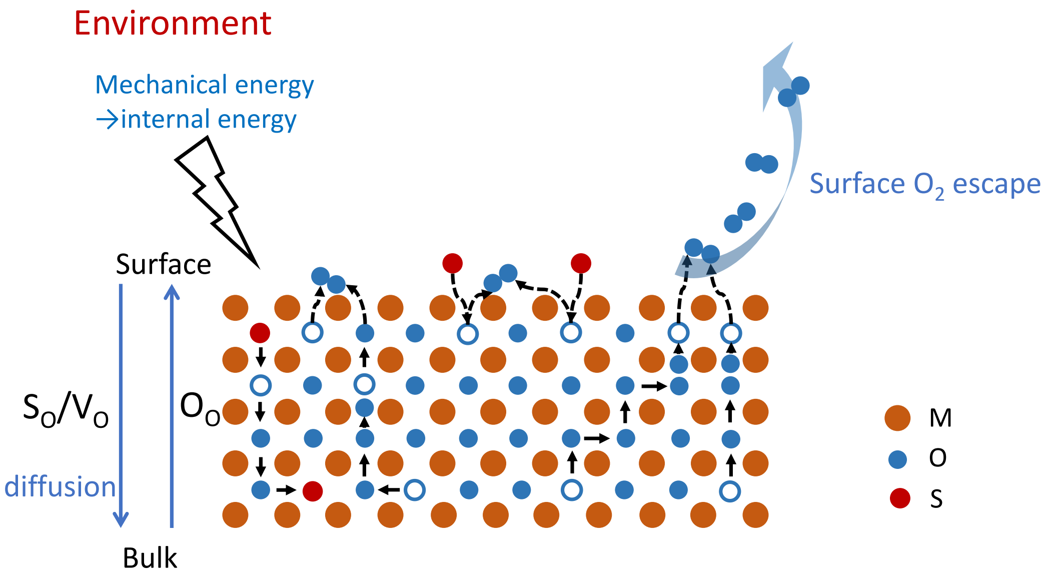

Figure S31. The synthesis mechanism of substitutional sulfur in mechanochemical process.

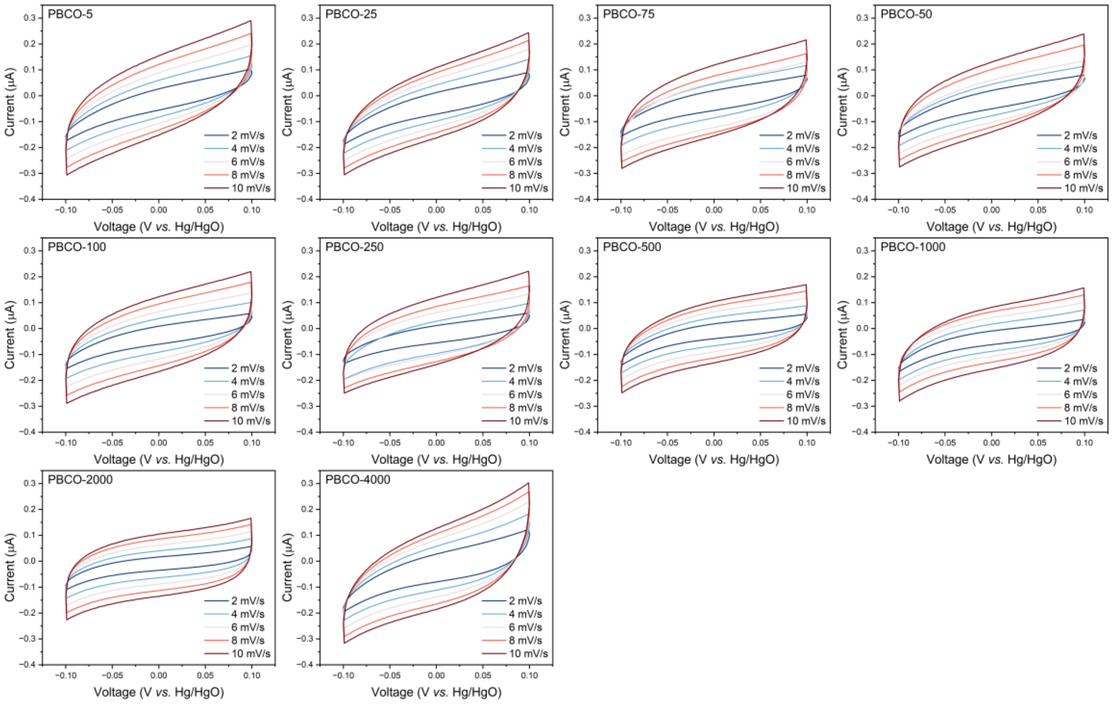

Figure S32. The cyclic voltammetry curves under different scanning rates of all PBCOs.

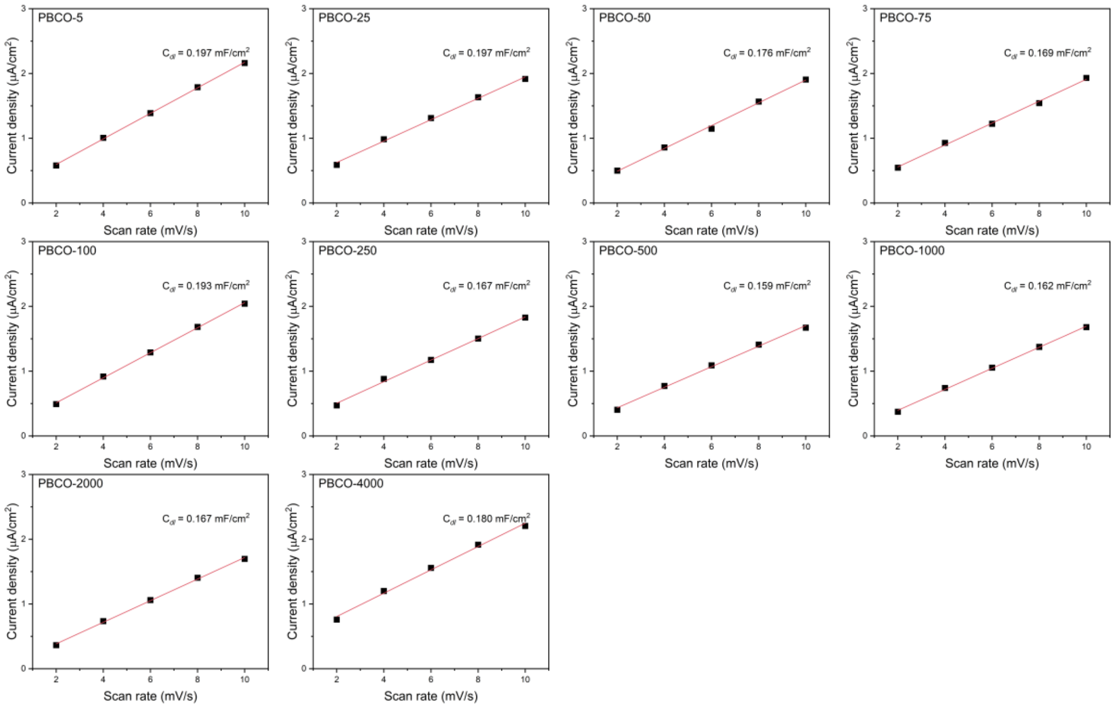

Figure S33. The electrochemical double-layer capacitance fitting curves of all PBCOs.

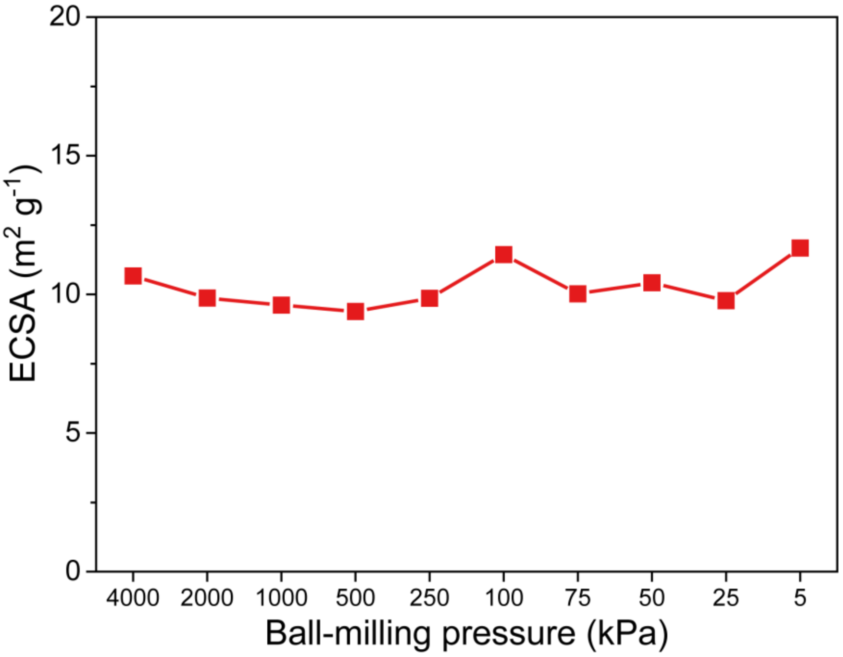

Figure S34. The electrochemical active surface area (ECSA) of PBCOs after ball-milling.

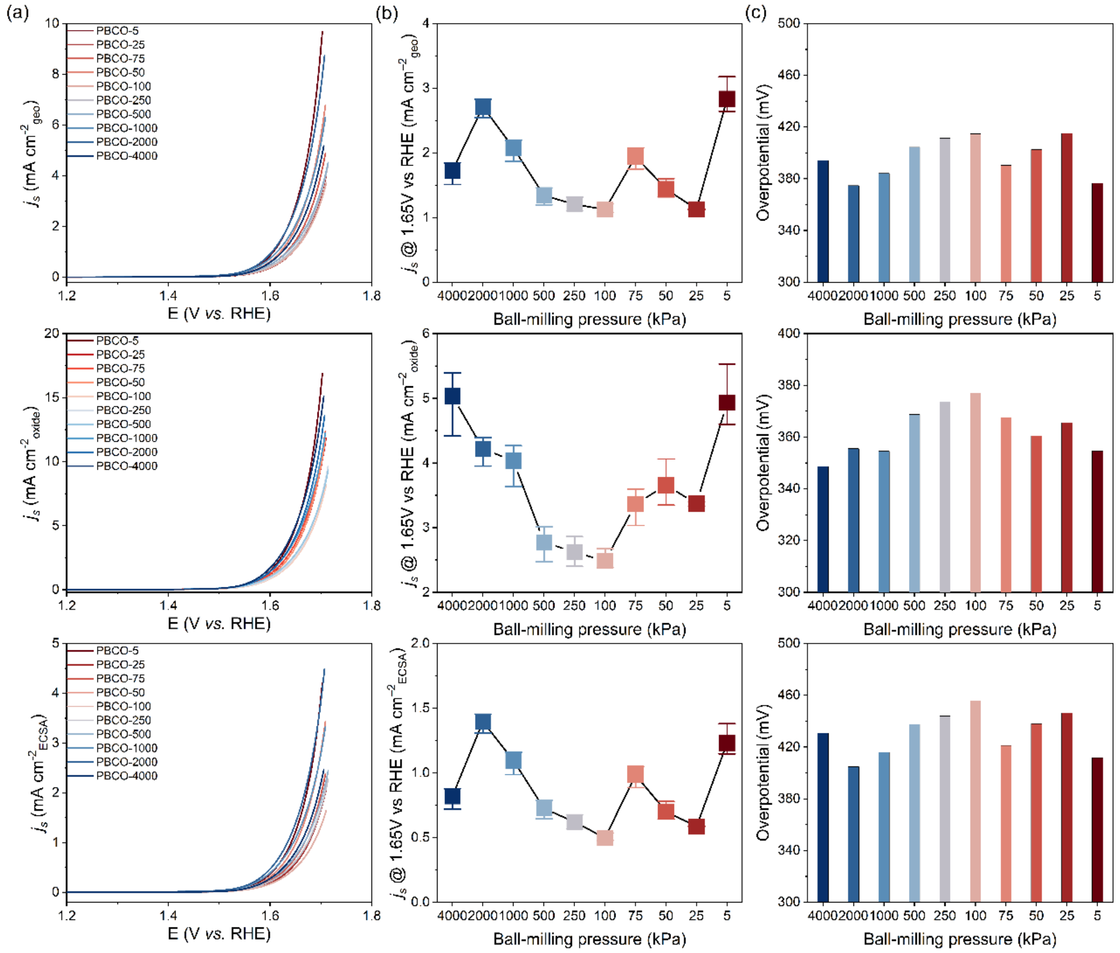

Figure S35. (a-c) Polarization curves of PBCOs from 2nd CV curves, the current density is normalized by the electrode geometric area (a), the BET surface area (b), and the ECSA (c), respectively. **(d-f)** Relationship between OER activities and ball-milling pressure of PBCOs from 2nd CV curves, the current density is normalized by the electrode geometric area (d), the BET surface area (e), and the ECSA (f), respectively. **(g-i)** Relationship between overpotential (1 mA cm^{-2}) and ball-milling pressure of PBCOs from 2nd CV curves, the current density is normalized by the electrode geometric area (g), the BET surface area (h), and the ECSA (i), respectively.

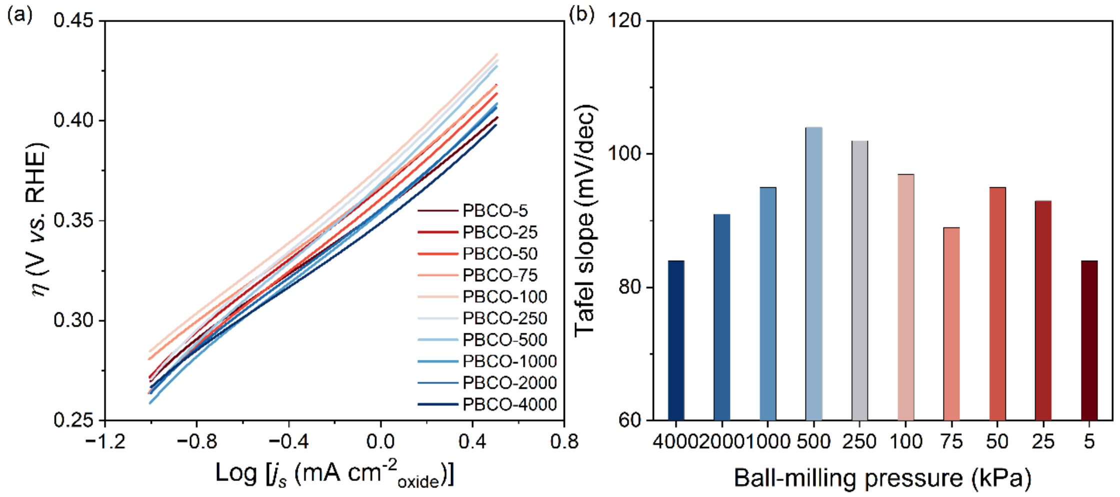

Figure S36. (a) Tafel curves of PBCOs from 2nd CV curves. **(b)** Relationship between Tafel slope and ball-milling pressure of PBCOs from 2nd CV curves.

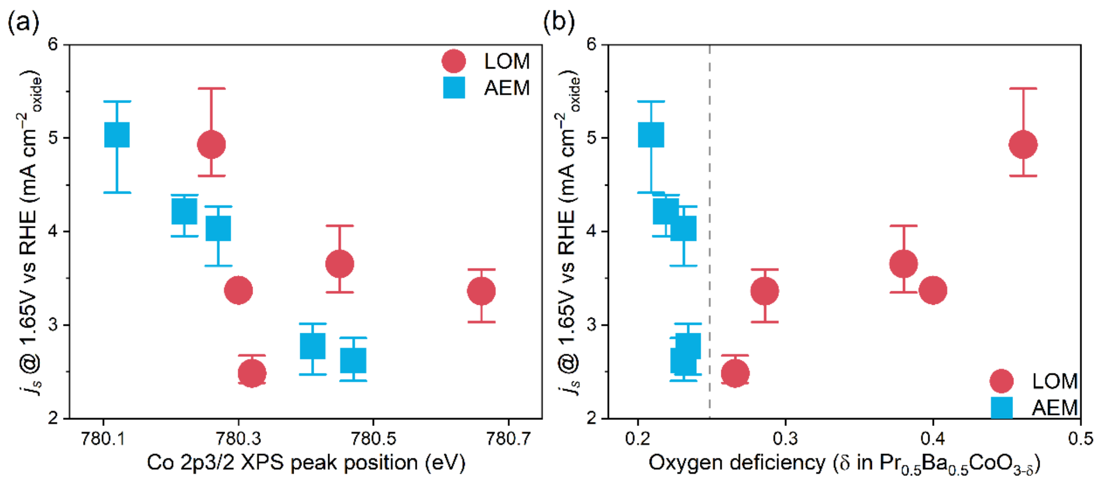

Figure S37. (**a**) Relationship between OER mechanisms and surface V_O concentration parameters. (**b**) relationship between OER mechanisms and bulk V_O concentration.

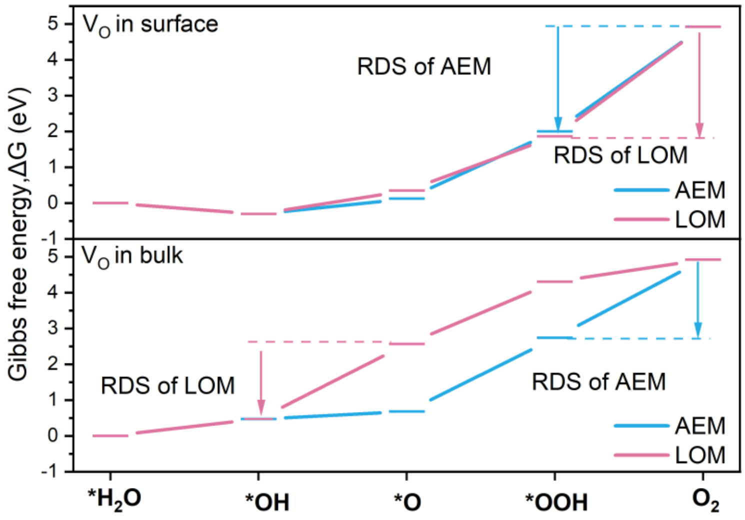

Figure S38. The calculation of the OER free energy and the rate-limiting step of surface and bulk V_O model.

Table S1. ICP results of PBCOs.

Ball-milling pressure (kPa)	Pr (mmol)	Ba (mmol)	Co (mmol)
**4000**	0.00838	0.00911	0.01733
**2000**	0.0071	0.00783	0.01449
**1000**	0.00901	0.0099	0.01884
**500**	0.00773	0.00834	0.01702
**250**	0.00783	0.00861	0.01635
**100**	0.00748	0.00795	0.01596
**75**	0.0078	0.00818	0.01639
**50**	0.00824	0.0087	0.01748
**25**	0.00776	0.0082	0.01656
**5**	0.00776	0.0082	0.01656

Table S2. ICP results for impurities of PBCO, PBCO-5, PBCO-100 and PBCO-4000.

Ball-milling pressure (kPa)	Co (mg/L)	Si (mg/L)	Fe (mg/L)	Ni (mg/L)
**blank**	0.0016	0.0560	0.0216	0.0032
**4000**	12.75	0.0515	0.0362	0.0028
**100**	13.37	0.0522	0.1077	0.0041
**5**	12.78	0.0511	0.0464	0.0028
**withou ball-milling**	12.12	0.0384	0.0134	0.0006

Table S3. BET specific surface area ($m^2\ g^{-1}$) of PBCOs.

**Ball-milling pressure (kPa)**	**BET specific surface area ($m^2\ g^{-1}$)**
Withou ball-milling	0.1668
4000	1.7364
2000	3.2656
1000	2.609
500	2.4534
250	2.3316
100	2.2946
75	2.9285
50	1.9999
25	1.6980
5	2.9140

Table S4. Calculated valence state of Co, oxygen defect contents (δ) and corresponding molecular formula in PBCOs. The valence states of Co were calculated based on XAFS data.

Ball-milling pressure (kPa)	valance state of Co	δ	molecular formula
**4000**	3.0824	0.209	$Pr_{0.5}Ba_{0.5}CoO_{2.791}$
**2000**	3.062	0.219	$Pr_{0.5}Ba_{0.5}CoO_{2.781}$
**1000**	3.038	0.231	$Pr_{0.5}Ba_{0.5}CoO_{2.769}$
**500**	3.032	0.234	$Pr_{0.5}Ba_{0.5}CoO_{2.766}$
**250**	3.036	0.232	$Pr_{0.5}Ba_{0.5}CoO_{2.768}$
**100**	2.968	0.266	$Pr_{0.5}Ba_{0.5}CoO_{2.734}$
**75**	2.927	0.286	$Pr_{0.5}Ba_{0.5}CoO_{2.714}$
**50**	2.741	0.38	$Pr_{0.5}Ba_{0.5}CoO_{2.620}$
**25**	2.7	0.4	$Pr_{0.5}Ba_{0.5}CoO_{2.600}$
**5**	2.578	0.461	$Pr_{0.5}Ba_{0.5}CoO_{2.539}$

Table S5. Lists the performance data of previously reported catalysts utilized in AWE cells.

Catalysts	Current density (A cm^{-2})	Cell voltage (V)	Temp (℃)	KOH	Durability (h)	Ref
NiFe-LDH || Pt foil	0.4	1.77	80	20 wt.%	100	10
NiFe-LDH/Ni_3S_2 || Raney Ni	0.8	1.908	90	30 wt.%	40	11
CoFeP || CoFeP	0.5	1.80	80	6 M	1500	12
(Fe,Ni)OOH || NiMoN	0.375	1.584	60	6 M	200	13
$Fe_{40}Ni_{40}P_{14}B_6$-72h || Pt/C	1	1.94	80	6 M	300	14
NFA900-ac || Raney Ni	0.5	1.867	80	5 M	1100	15
Pt@fNi$(OH)_2$ || Ni	0.4	2.11	65	30 wt.%	600	16
Co, Mo-NiFe LDH || Raney Ni	0.5	1.91	85	30 wt.%	400	17
(Ni, Fe)$_3S_2$ /NFF || (Ni, Fe)$_3S_2$ /NFF	0.6	1.93	80	30 wt.%	600	18
Ni-Mo || Ni-Fe	1	2.1	80	30 wt.%	-	19
Ni-Fe || Raney Ni	0.6	2	80	30 wt.%	100	20
Ni-MoO_2-450NWs/ CC	1	2.42	80	30 wt.%	40	21
60Fe/NF || NiMo	0.4	3.4	60	6 M	100	22
NiMo@NM||NiFe@ NM	0.4	1.83	70	6 M	50	23
NiFe@CuO|| NiFe@CuO	0.1	1.5	80	6 M	20	24
NiFeMo@NF|| NiFeMo@NF	0.2	1.81	90	30 wt.%	672	25
NCP/NC/Ni || Ni	0.7	2	85	30 wt.%	720	26
FeCoCrCuOx@CF||	0.5	2.17	80	6 M	70	27

$Fe_{2.5}Co_{2.5}Ni_{10}O_yH_z$ @NFF						
NiFeW@RN || NiFeW@RN	0.1	1.48	80	6 M	100	28
NiFeOxHy-PN || Raney Ni	0.4	1.86	85	30 wt.%	12	29
This work	1	1.96	80	30 wt.%	300	This work

References

[1] Suntivich J, Gasteiger H A, Yabuuchi N, et al. Electrocatalytic measurement methodology of oxide catalysts using a thin-film rotating disk electrode. *J Electrochem Soc* 2010; 157: B1263.

[2] Kresse G, Furthmüller J. Efficient iterative schemes for *Ab Initio* total-energy calculations using a plane-wave basis set. *Phys Rev B* 1996; 54: 11169-11186.

[3] Kresse G, Furthmüller J. Efficiency of *Ab Initio* total energy calculations for metals and semiconductors using a plane-wave basis set. *Comput Mater Sci* 1996; 6: 15-50.

[4] Perdew J P, Chevary J A, Vosko S H, et al. Atoms, molecules, solids, and surfaces: applications of the generalized gradient approximation for exchange and correlation. *Phys Rev B* 1992; 46: 6671-6687.

[5] Monkhorst H J, Pack J D. Special points for brillouin-zone integrations. *Phys Rev B* 1976; 13: 5188-5192.

[6] Zhao B, Zhang L, Zhen D, et al. A tailored double perovskite nanofiber catalyst enables ultrafast oxygen evolution. *Nat Commun* 2017; 8: 14586.

[7] Kushima A, Yip S, Yildiz B. Competing strain effects in reactivity of $LaCoO_3$ with oxygen. *Phys Rev B* 2010; 82: 115435.

[8] Wang Y, Cheng H-P. Oxygen Reduction Activity on Perovskite Oxide Surfaces: A comparative first-principles study of $LaMnO_3$, $LaFeO_3$, and $LaCrO_3$. *J Phys Chem C* 2013; 117: 2106-2112.

[9] Kim J, Shih P-C, Tsao K-C, et al. High-performance pyrochlore-type yttrium ruthenate electrocatalyst for oxygen evolution reaction in acidic media. *J Am Chem Soc* 2017; 139: 12076-12083.

[10] Liu W, Ding X, Cheng J, et al. Inhibiting dissolution of active sites in 80 ℃ alkaline water electrolysis by oxyanion engineering. *Angew Chem Int Ed* 2024; 136, e202406082.

[11] Wu W, Wang Y, Song S, et al. Built-in electric field in freestanding hydroxide/sulfide heterostructures for industrially relevant oxygen evolution. *Angew Chem Int Ed* 2025; 137: e202504972.

[12] Wang X, Zhang Z, Zhang H, et al. Crystalline/amorphous phosphide heterostructures with built-in electric fields for efficient and long-term industrial-scale alkaline water electrolysis. *Adv Funct Mater* 2025; e14137.

[13] Wu L, Ning M, Xing X, et al. Boosting oxygen evolution reaction of (Fe, Ni)OOH via defect engineering for anion exchange membrane water electrolysis under industrial conditions. *Adv Mater* 2023; 35: 2306097.

[14] Zhu Z F, Gu J, Zhao W, et al. Multilayer catalysts with glass-crystal dual-phase heterostructures for exceptional alkaline water electrolysis under industrial conditions. *Nano Energy* 2025; 111357.

[15] Zhang H, Jing C, Xiong X, et al. Porous NiFe alloy with self-repair surface layer for superior industrial water electrolysis. *Appl Catal B-Environ Energy* 2025; 125697.

[16] Ma J, Zhu X, Li Y, et al. Highly durable Pt@fNi$(OH)_2$Ni cathode prepared by in situ electrodeposition method for alkaline water electrolysis. *Electrochimi Acta* 2024; 494: 144454.

[17] Zhao Y, Wen Q, Huang D et al. Operando reconstruction toward dual-cation-defects Co-containing NiFe oxyhydroxide for ultralow energy consumption industrial water splitting electrolyzer. *Adv Energy Mater* 2023; 13: 2203595.

[18] Bai X, Zhang M, Shen Y et al. Room-temperature, meter-scale synthesis of heazlewoodite-based nanoarray electrodes for alkaline water electrolysis. *Adv Funct Mater* 2024;

34: 2400979.
[19] Liao Y, Deng G, Ding L, et al. A thin and flexible composite membrane with low area resistance and high bubble point pressure for advanced alkaline water electrolysis. *J Membr Sci* 2024; 689: 122182.
[20] Kumar S S, Ramakrishna S U B, Krishna S V, et al. Synthesis of titanium (IV) oxide composite membrane for hydrogen production through alkaline water electrolysis. *S Afr J Chem Eng* 2018; 25: 54.
[21] Ren B, Li D, Jin Q, et al. Integrated 3D self-supported Ni decorated MoO_2 nanowires as highly efficient electrocatalysts for ultra-highly stable and large-current-density hydrogen evolution. *J Mater Chem A* 2017; 5: 24453-24461.
[22] Zhuo Y, Liu D, Qiao L, et al. Ultrafast room-temperature synthesis of large-scale, low-cost, and highly active Ni-Fe based electrodes toward industrialized seawater oxidation. *Adv Energy Mater* 2023; 13: 2301921.
[23] Zhu L, Fang Q Y, Liu S T, et al. Two closed-loop nickel-based catalysts for use in alkaline water electrolysis under industrial conditions. *J Solid State Electr* 2024; 28: 3915-3927.
[24] Ma X, Chen F, Yang X, et al. Construction of three-dimensional array nanorod heterojunction catalysts with high efficiency and stability in industrial environments. *Fuel* 2025; 380: 133135.
[25] Frederiksen M L, Oglou R C, Lauritsen J V, et al. Electrocatalytic and structural investigation of trimetallic NiFeMo bifunctional electrocatalyst for industrial alkaline water electrolysis. *Electrochimi Acta* 2024; 482: 143988.
[26] Chen C, Zhou J, Shen J, et al. Hierarchical NiCoP/NiCo architecture on Ni mesh boosts hydrogen production under industrial alkaline conditions. *Chem Eng J* 2024; 484: 149456.
[27] Liu C, Bai H, Feng J, et al. Electrodeposited ternary metal (oxy)hydroxide achieves highly efficient alkaline water electrolysis over 1000 h under industrial conditions. *Carbon Energy* 2025; e684.
[28] Wang Y, Chen F, Zhao Z, et al. W-doped hydrangea-like NiOOH/FeOOH catalyst for achieving efficient industrial alkaline water splitting. Int. *J Hydrogen Energ* 2025; 175: 151465.
[29] Zhong B, Kuang P, Wang L, et al. Hierarchical porous nickel supported $NiFeO_xH_y$ nanosheets for efficient and robust oxygen evolution electrocatalyst under industrial condition. *Appl Catal B-Environ Energy* 2021; 299: 120668.
